# Increasing Expression of *PnGAP* and *PnEXPA4* Provides Insights Into the Enlargement of *Panax notoginseng* Root Size From *Qing* Dynasty to Cultivation Era

**DOI:** 10.3389/fpls.2022.878796

**Published:** 2022-05-20

**Authors:** Mu-Yao Yu, Zhong-Yi Hua, Pei-Ran Liao, Han Zheng, Yan Jin, Hua-Sheng Peng, Xiu-Ming Cui, Lu-Qi Huang, Yuan Yuan

**Affiliations:** ^1^State Key Laboratory Breeding Base of Dao-di Herbs, National Resource Center for Chinese Materia Medica, China Academy of Chinese Medical Sciences, Beijing, China; ^2^School of Traditional Chinese Medicine, Guangdong Pharmaceutical University, Guangzhou, China; ^3^Faculty of Life Science and Technology, Kunming University of Science and Technology, Kunming, China

**Keywords:** root size, cultivation, GPI-anchored, expansin, *Panax notoginseng*, cell wall

## Abstract

Root size is a key trait in plant cultivation and can be influenced by the cultivation environment. However, physical evidence of root size change in a secular context is scarce due to the difficulty in preserving ancient root samples, and how they were modified during the domestication and cultivation stays unclear. About 100 ancient root samples of *Panax notoginseng*, preserved as tribute in the Palace Museum (A.D. 1636 to 1912, *Qing* dynasty), provided an opportunity to investigate the root size changes during the last 100 years of cultivation. The dry weight of ancient root samples (~120 *tou* samples, *tou* represents number of roots per 500 g dry weight) is 0.22-fold of the modern samples with the biggest size (20 *tou* samples). Transcriptome analysis revealed that *PnGAP* and *PnEXPA4* were highly expressed in 20 *tou* samples, compared with the 120 *tou* samples, which might contribute to the thicker cell wall and a higher content of lignin, cellulose, and callose in 20 *tou* samples. A relatively lower content of dencichine and higher content of ginsenoside Rb_1_ in 20 *tou* samples are also consistent with higher expression of ginsenoside biosynthesis-related genes. PnPHL8 was filtrated through transcriptome analysis, which could specifically bind the promoters of *PnGAP, PnCYP716A47*, and *PnGGPPS3*, respectively. The results in this study represent the first physical evidence of root size changes in *P. notoginseng* in the last 100 years of cultivation and contribute to a comprehensive understanding of how the cultivation environment affected root size, chemical composition, and clinical application.

## Introduction

Plant root size is a key trait for improving water and nitrogen uptake efficiency. In cultivation, temperature (Teskey and Hinckley, 2010; Chathurika et al., 2018), precipitation (Ba Rraclough et al., 2010; Ghaffari et al., 2021), light transmittance (Cheon et al., 2004; Kuang et al., 2015), fertilization (Ba Rraclough et al., 2010; Goodsman et al., 2010), and agrotype (Hiroyoshi et al., 2004; Chen, 2017) can lead to deformation of plant root phenotype. Except for the environmental factors, the genotype is the main factor determining the root size, and a number of quantitative trait loci (QTL) or genes associated with root size have been identified (Jeong et al., 2013; Tamirisa et al., 2014; Yao et al., 2014; Cheng et al., 2016; Ding et al., 2016). It was reported that glycosylphosphatidylinositol (GPI)-anchored protein (*GAP*) has diverse function on root architecture, by affecting cell wall architecture (Macmillan et al., 2010), cell elongation (Niu et al., 2018), cytoderm thickness, and content of lignin, cellulose, and callose (Liu, 2009; Bundy et al., 2016; Zhao et al., 2020). High temperature decreased the expression of *OsGAP18*, leading to a thinner cell wall (Zhao et al., 2020), while several *GAP*s were prominently upregulated during cold acclimation (Daisuke et al., 2016) and nitrogen supply (Engelsberger and Schulze, 2012). Expansin (EXP) also plays significant role in root architecture, and the expression of EXPAs could be induced under cold acclimation, water stress, and higher application of fertilizer (Bian, 2006; Kozbial et al., 2010; Li et al., 2013, 2019b; Sun, 2013; Han et al., 2014; Ren et al., 2019).

*Panax notoginseng* is a popular functional food and traditional medicine, whose benefits are considered to be represented by the root size and ginsenoside content. The main root of *P. notoginseng* cultivated at higher altitude is significantly larger (Zheng et al., 2014). Root diameter is directly proportional to light transmittance within limit (Kuang et al., 2014; Wang et al., 2018a), and the application of phosphorus and potassium can increase root weight and promote root thickening (Wang et al., 2008; Zhang et al., 2008). A suitable soil texture possesses great fertilizer preserving capability and abundant mineral elements, promoting the growth of root system (Cui et al., 2005; Li et al., 2016).

Cultivation also affects the content of ginsenosides and amino acids, as well as the transcriptional level of corresponding biosynthetic genes in *P. notoginseng*. High precipitation inhibits the accumulation of total ginsenosides, while low temperature induced upregulation of *HMGR, SS*, and *SE* and increased the ginsenoside content (Liu et al., 2016b; Ma et al., 2021). Increasing the concentration of potassium, nitrogen, magnesium, and calcium brings a remarkable boost to the ginsenoside content, in a certain range (Konsler et al., 1990; Yu et al., 2001). As a key enzyme in dencichine biosynthesis, activity of serine acetyltransferase (*SAT*) can be decreased by drought stress (Ahmad et al., 2016; Yang et al., 2021).

The cultivation of *P. notoginseng* can be tracked back to *Jiaqing* year in *Qing* dynasty, which is around A.D. 1800 (Wu, 1963). The Palace Museum (Beijing, China) has abundant and well-preserved *P. notoginseng* samples from *Qing* dynasty, and those ancient root samples were tributes from Guangxi or Yunnan provinces to the emperor, which represented the best quality of *P. notoginseng* at that time. The cultivation environments of *P. notoginseng* showed a great improvement toward planting temperature and humidity (Hua, 1967; He and Deng, 1981; China Association of Chiniese Medicine., 2019), light transmittance (Chen, 1958; Huang et al., 2007; China Association of Chiniese Medicine., 2019), soil type (He and Deng, 1981; China Association of Chiniese Medicine., 2019), and fertilizer application (Chen, 1958; He and Deng, 1981; China Association of Chiniese Medicine., 2019) during past 100 years, with root size also showing an enlargement since 1950s (Jin et al., 1996; Xu, 2016). Tributes from *Qing* dynasty ought to be an excellent material to investigate how long cultivation influenced root development.

In this study, we compared the root length, diameter, and weight of *P. notoginseng* from the Palace Museum (Beijing, China) and modern market and performed detailed RNA-seq, UPLC-QQQ-MS, and desorption electrospray ionization analyses of two types of root size (SRW samples, 120 *tou* with small root weight, representing substitutes for *Qing* dynasty tribute; and LRW samples, 20 *tou* with large root weight, representing for highest benefits in modern market). We used these datasets to explore how domestication and cultivation lead to deformation of *P. notoginseng* root size and to investigate the gene-level regulatory mechanisms that control a better-architected cell wall obtained in LRW samples, providing guidance on artificial cultivation of *P. notoginseng*.

## Materials and Methods

### Plant Materials

About 100 root samples of *P. notoginseng* in *Qing* dynasty were obtained from The Palace Museum, Beijing. Six types of dry root samples with different sizes (20 *tou*, 40 *tou*, 60 *tou*, 80 *tou*, 120 *tou*, and >120 *tou, tou* represents the number of roots per 500 g dry weight) were purchased from Anhui Tienho Herbal Source Company. Fresh root samples were collected in Wenshan, Yunnan Province, and stored at −80°C (Zheng et al., 2017). According to the drying rate (Xu, 2016), 21 fresh roots of *P. notoginseng* were divided into two groups: (1) Seven LRW samples that have fresh weight of 14.27 ± 2.54 g and are equivalent to 20 *tou* samples, and (2) 14 SRW samples that have fresh weight of 5.17 ± 0.62 g and are equivalent to 120 *tou* samples (Supplementary Table 1).

### Analysis of RNA Sequencing Profiles

Raw data from transcript database of *P. notoginseng* (Zheng et al., 2017) were used for further analysis. Clean data were obtained by removing reads with low quality from raw data. Filtered reads were aligned to the *P. notoginseng* genome (Jiang et al., 2020) using STAR (Valencia, 2014) and counted using RSEM (Dewey and Li, 2011). Differentially expressed genes (DEGs) were obtained by comparing the gene expression in LRW and SRW *P. notoginseng* using DESeq2 (Love et al., 2014). Genes with padj below 0.05 and log2 (fold change) >1 were considered as DEGs.

### Microscope Observation

Root material was cut into 3-mm tissue samples and dehydrated with a gradient of 70, 80, 95, and 100% of ethanol. The tissue was treated with xylene twice for cell permeabilization and then was soaked and embedded with paraffin. After that, the tissue was cut into 5-μm slice, which was heated and dewaxed in water. Microsection was observed using an Olympus BX51 microscope. The diameter of vessel and the thickness of cell wall were measured using DP2-BSW software. The diameters of vessels in one field of view were measured and averaged, and six fields of view of each five biological replicates were obtained for *t*-test. Measurement of cell wall thickness in each tissue was same as that of the vessel diameter, and the thickness of 10 cells in one field of view was measured and averaged.

### Cell Wall Component Measurement

Sulfuric acid hydrolysis method was used to determine the lignin content (Xiong et al., 2005; Chen et al., 2010). LRW and SRW samples were freeze-dried and ground into powder. About 100 mg of powder was weighed and extracted with 1% acetic acid solution twice. The precipitate was soaked in a mixture of ethanol and diethyl ether (1:1) for three times and evaporated to residue. About 3 mL of 72% (w/v) sulfuric acid was mixed up with the precipitate and stood for 16 h at room temperature. Then, 10 mL of distilled water was added and placed in boiling water bath for 5 min. After cooling, 5 mL of distilled water and 0.5 mL of 10% (w/v) barium chloride solution were added, and the residue was washed with distilled water subsequently. About 10 mL of 10% (w/v) sulfuric acid solution and 10 mL of 0.1 mol·L^−1^ potassium dichromate solution were added to the residue and heated in boiling water for 15 min. The supernatant after cooling was transferred to flask and then mixed up with 5 mL of 20% (w/v) KI solution and 1 mL of 0.5% (w/v) starch solution for titration. The titrant was 0.2 mol·L^−1^ of sodium thiosulfate.

Determination of cellulose was performed using enzyme-linked immunosorbent assay (ELISA), according to the manufacturer's instructions (Jiangsu Jingmei Biological Technology Co. Ltd., China. Item number, JM-110113P1). Homogenized LRW and SRW samples were extracted in 900-μL PBS buffer (pH 7.4), and then, supernatant was obtained by centrifugation at 2,000 rpm for 20 min. Microtitration plates were coated with purified cellulose antibody. After 5 times dilution, the sample solution was added to coated micropore, which subsequently bound with HRP-labeled cellulose antibody at 37°C for 30 min. Tetramethylbenzidine (TMB) was added as substrate and then incubated in the dark at 37°C for 10 min. The reaction was terminated using 1 mol·L^−1^ of sulfuric acid, and the absorbance value was determined at 450 nm. Calibration curve was obtained using cellulose standard solution of 400, 200, 100, 50, and 25 ng·L^−1^.

Callose determination was performed according to the published method (Khle et al., 1985) with some modification. Briefly, 100 mg of fresh plant materials was washed with ethanol for three times to eliminate autofluorescence, ground in liquid nitrogen, and extracted with 1 mL of 1 mol·L^−1^ NaOH at 80°C for 15 min. After centrifugation (10,000 × g, 15 min), the supernatant was mixed with 0.1% (w/v) aniline blue to produce a violet-red color. Then, 1 mol·L^−1^ of glycine/NaOH buffer (pH 9.5) was added and incubated at 50°C for 20 min, and then at room temperature for 20 min. Fluorescence was recorded using a HITACHI F-7000 spectrofluorometer (Tokyo, Japan) with the following parameters: excitation wavelength of 400 nm, emission wavelength of 510 nm, and slit width of 10 nm. Calibration curve was obtained using β-1,3-glucan in 1 mol·L^−1^ of NaOH.

### qRT-PCR Analysis

Total RNA was extracted from root of *P. notoginseng*, using the Plant RNA Purification Reagent (Invitrogen, USA), according to the manufacturer's instructions. qRT-PCR was performed on a LightCycler 480 Real-Time PCR System (Roche Diagnostics, Basel, Switzerland), with primers listed in Supplementary Table 2.

### Determination of Ginsenosides Using UPLC-QTRAP-MS/MS

The ginsenoside content was measured as described previously (Liu et al., 2020), with some modifications. *P. notoginseng* samples were first ground into powder, and 0.1 g powder was weighed accurately into 5-mL centrifuge tubes and extracted with 2 mL of 70% ethanol solution. The tubes were then sonicated for 30 min at room temperature. The supernatant was collected after being centrifuged for 10 min at 13,000 g. The test solution was obtained by filtrating the supernatant through 0.22-μm Millipore filter.

UPLC was performed on a Waters ACQUITY UPLC I-Class system. Chromatographic separations were performed on a ACQUITY BEH C18 column (2.1 × 100 mm, 1.7 μm) with a flow rate of 0.5 mL·min^−1^ at 40°C. The mobile phase was composed of 0.1% of formic acid–acetonitrile (A) and 0.05% of formic acid–water (B). Gradient elution program was as follows: 0–0.5 min, 20% A; 0.5–3 min, 20–80% A; 3–3.1 min, 80–98% A; 3.1–5 min, 98% A; 5–5.1 min, 98–20% A; 5.1–8 min 20% A. The injection volume was 1 μL for each sample.

Mass analysis was performed on a ABSCIEX 6500 QTRAP mass spectrometer. Mass spectrometer was performed in a positive ion mode using multiple reaction monitoring (MRM) mode. Optimized MS/MS parameters of saponins are shown in Supplementary Table 3. Ion spray voltage was set at 5500 eV, and turbo spray temperature was 550°C. Both gas 1 and gas 2 were set at 50 psi.

### Quantitation of Dencichine Using UPLC-UV-MS

Determination of dencichine was performed as reported with some modification (Ju et al., 2015). About 0.1 g of root power was added with 5 mL of 70% methanol solution, and the mixture was sonicated for 2 h subsequently. The supernatant was obtained through centrifugation at 12,000 × g for 15 min and then diluted with 70% of methanol for 10 times before quantification. Dencichine was detected on a Waters ACQUITY UPLC I-Class system, equipped with a PDA detector under a UV wavelength of 213 nm. An ACQUITY BEH C18 column (2.1 × 100 mm, 1.7 μm) was used for separation, with a flow rate of 0.3 mL·min^−1^. Mobile phase A consisted of 0.05% phosphoric acid in water, while mobile phase B was methanol, and an isocratic elution of 53% A was used. Injection of samples was 1 μL.

### Yeast One-Hybrid Assay

Y1H assay was performed as described previously (Zheng et al., 2021). The sequences of *proPnEXPA4, proPnGGPPS3, proPnFPS, proPnCYP716A47, proPnCYP716A53v2*, and *proPnGAP* were nested PCR-amplified according to genomic sequences (Jiang et al., 2020). The probable binding domains of promoters were inserted into pAbAi vector as baits and were integrated into Y1HGold, and then, the minimal inhibitory concentration of aureobasidin (AbA) was tested on SD/-Uracil (Ura) plates. A pGADT7-*PnPHL8* recombinant plasmid was synthesized by RuiBiotech Co. Ltd., as prey. The pGADT7-*PnPHL8* construct and a blank pGADT7 were introduced into bait reporter strains, while blank pGADT7 was used as control. Positive transformants were selected on SD/-Leucine (Leu)/-Ura plates with an appropriate concentration of AbA. Primers used for promotor nested PCR amplification and bait construction are listed in Supplementary Table 2.

### Electrophoretic Mobility Shift Assay

EMSA was performed as described previously (Zheng et al., 2021). The full-length cDNA of *PnPHL8* was first cloned into a pMAL-c2x vector and then transformed into a Rosetta (DE3) competent cell. Prokaryotic expression was performed at 20°C for 12 h, and then, recombinant protein was purified using Amylose Resin High Flow (NEB, Ipswich, MA, USA). EMSA was performed according to the manufacturer's instructions, using chemiluminescent EMSA kit (Beyotime, Item number, GS009). Primers and probes are listed in Supplementary Table 2.

## Results

### Transition of Root Size of *P. notoginseng* From *Qing* Dynasty to Modern Times

**Figure 1:**
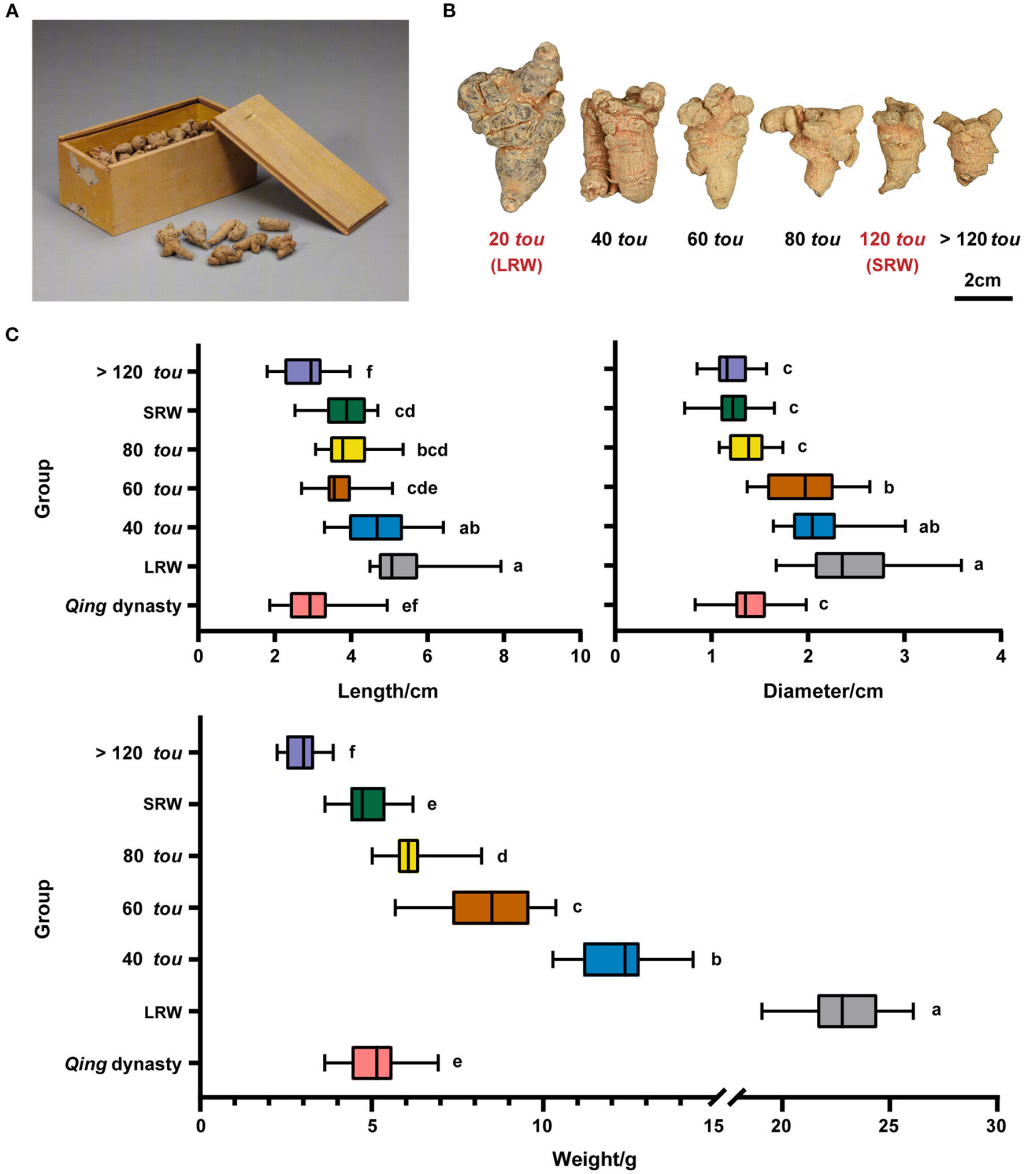
Morphological features of *P. notoginseng* in *Qing* dynasty and modern times. **(A)** *P. notoginseng* in *Qing* dynasty, relic number, Gu00172482-8/9. **(B)** *P. notoginseng* of different root sizes (20 *tou*, 40 *tou*, 60 *tou*, 80 *tou*, 120 *tou*, and >120 *tou*; *tou* is the number of roots per 500 g). **(C)** Length, diameter, and weight of *P. notoginseng* in *Qing* dynasty (*n* = 30) and in modern times (*n* = 20). Data are shown in box plot, and the different letters indicate values that vary significantly at *P* < 0.05 (one-way ANOVA).

To investigate the morphological difference between *Qin*g dynasty tributes and modern commodities, root length, diameter, and root weight of ancient samples (Figure 1A) from the Palace Museum and six types of root samples (20 *tou*, 40 *tou*, 60 *tou*, 80 *tou*, 120 *tou*, and >120 *tou*) bought from modern market (Figure 1B) were measured. The root of *P. notoginseng* in *Qing* dynasty had a similar diameter and dry weight with 120 *tou* samples, while the length of ancient samples was relatively shorter than the 120 *tou* samples (Figure 1C).

After 1950s, artificial cultivation techniques, for example, the application of chemical fertilizers, reduced light transmittance and temperature has been applied to the cultivation of *P. notoginseng* (Supplementary Table 4), and the biggest root weight increased from 6.25 to 12.5 g in 1950s (Jin et al., 1996) to 25 g at present (Liu et al., 2016a; Xu, 2016). The dry weight of *Qing* root samples is around 5.17 g, which is 0.22-fold of the biggest of modern samples (20 *tou* samples), suggesting that a transition in root size of *P. notoginseng* has occurred following the change in cultivation practices and environment among *Qing* dynasty, 1950's, and modern time.

### Higher Expression of GPI-Anchored Protein and Thicker Cell Wall in Root With Larger Weight

To further investigate the probable molecular mechanism behind the transition of root size, transcriptome sequencing was performed on 21 *P. notoginseng* root samples with two types of root size (LRW and SRW). A total of nine DEGs were identified between LRW and SRW samples, and the transcriptional levels of eight genes, namely a MYB-CC transcriptional factor, AUX/IAA, DNA ligase, ceramide glucosyltransferase, L-type lectin-domain containing receptor kinase, berberine bridge enzyme-like (*BBE*), β-1,3-galactosyltransferase (*GALT*), and E3 ubiquitin-protein ligase (*RIE*), were decreased in LRW samples (Table 1). Among the nine DEGs, only *BBE* was enriched into phenylpropanoid biosynthesis pathway in KEGG analysis, while no DEGs were enriched in GO analysis.

**Table 1:** Identification of nine DEGs in *P. notoginseng* of different sizes.

**ID**	**Description**	**FPKM**	
		**LRW**	**SRW**
PN010249	DNA ligase	5.08	46.60
PN012351	Berberine bridge enzyme-like 8 (*BBE*)	3.52	41.78
PN018218	L-type lectin-domain containing receptor kinase S4	0.05	1.03
PN023854	Uncharacterized GPI-anchored protein	5.99	1.91
PN024060	Auxin-responsive protein (*IAA7*)	20.94	79.16
PN024675	β-1,3-galactosyltransferase (*GALT1*)	0.11	1.95
PN024679	MYB family transcription factor (*PHL8*)	1.32	5.16
PN025758	Ceramide glucosyltransferase 3	1.82	4.96
PN035878	E3 ubiquitin protein ligase (*RIE1*)	1.21	5.32

**Figure 2:**
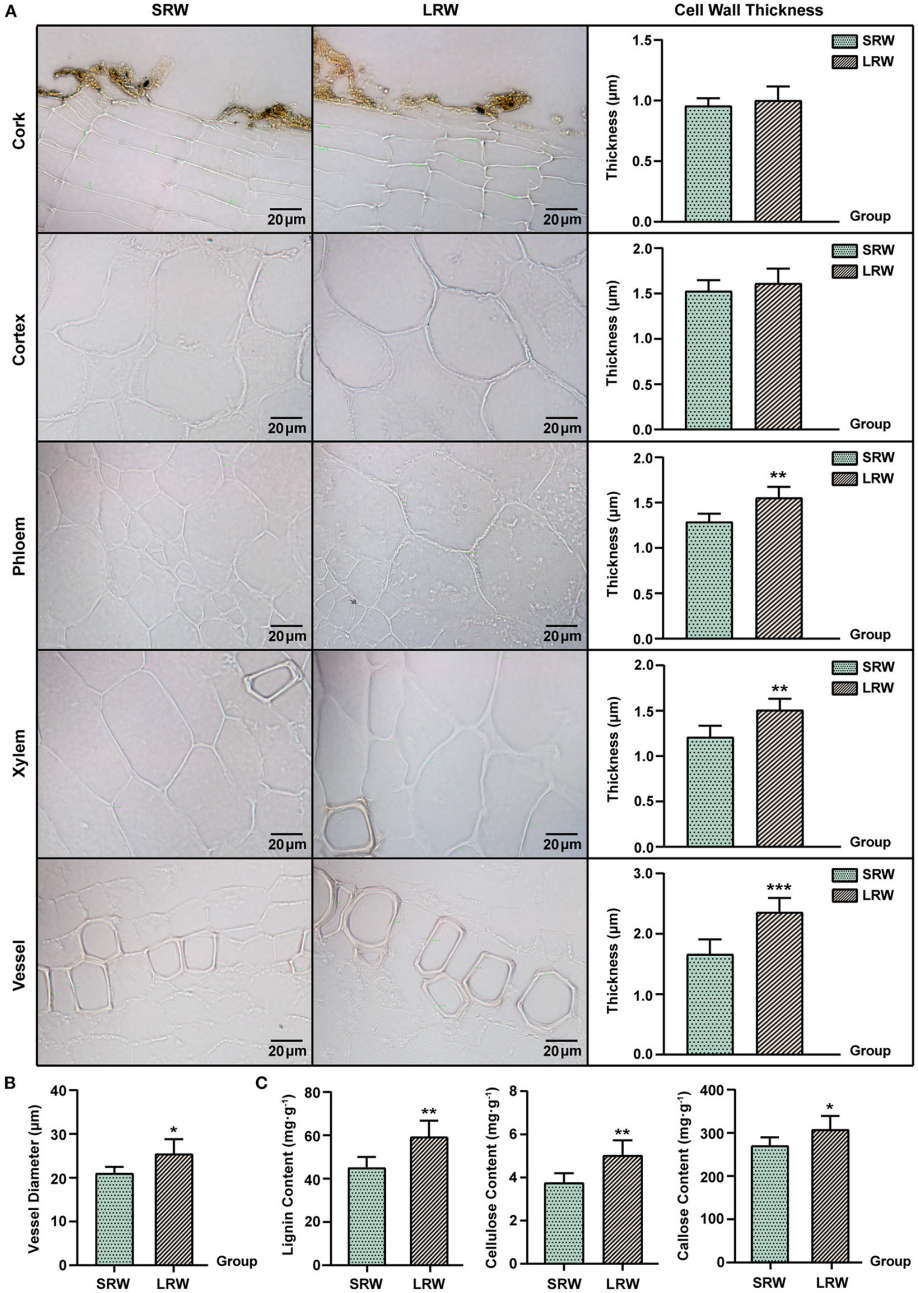
Different tissues and cell morphology in *P. notoginseng*. SRW was an abbreviation of small root weight, while LRW represented large root weight. **(A)** Microscope morphology of different tissues including cork, cortex, phloem, xylem, and vessel, and comparison of the cell wall thickness. **(B)** Comparison of the vessel diameter of *P. notoginseng* with different root weights. **(C)** Determination of lignin and callose in *P. notoginseng* with different root weights. The thickness of 10 cell walls in one field of view were measured and averaged, and then in accordance with this, a total of six fields of view from five biological replicates were obtained for *t*-test. Measurement of the vessel diameters was same as that of the cell wall thickness, while the diameter of seven vessels was measured and averaged. Asterisks denote Student's t-test significance: *P < 0.05 and **P < 0.01.

The expression of an uncharacterized GPI-anchored protein (GAP) was upregulated in LRW, with a 3.14-fold increase, compared with that in SRW. *GAP*s were reported to be associated with cell wall architecture (Macmillan et al., 2010; Niu et al., 2018), so we speculated that higher expression of *PnGAP* in LRW samples may lead to the thicker cell wall. We then measured the structure and component of cell wall in *P. notoginseng* root. Compared with SRW samples, cell wall in phloem and xylem of LRW samples was significantly thicker. An extremely significantly thicker cytoderm of vessel was detected in LRW, which was 1.41-fold of that in SRW. There was no remarkable difference of the cell wall thickness in cork and cortex between LRW and SRW samples (Figure 2A). In addition, LRW samples possessed a prominently larger vessel width than SRW samples (Figure 2B). As major components of cell walls, the content of lignin, cellulose, and callose was significantly higher in LRW samples (Figure 2C).

**Figure 3:**
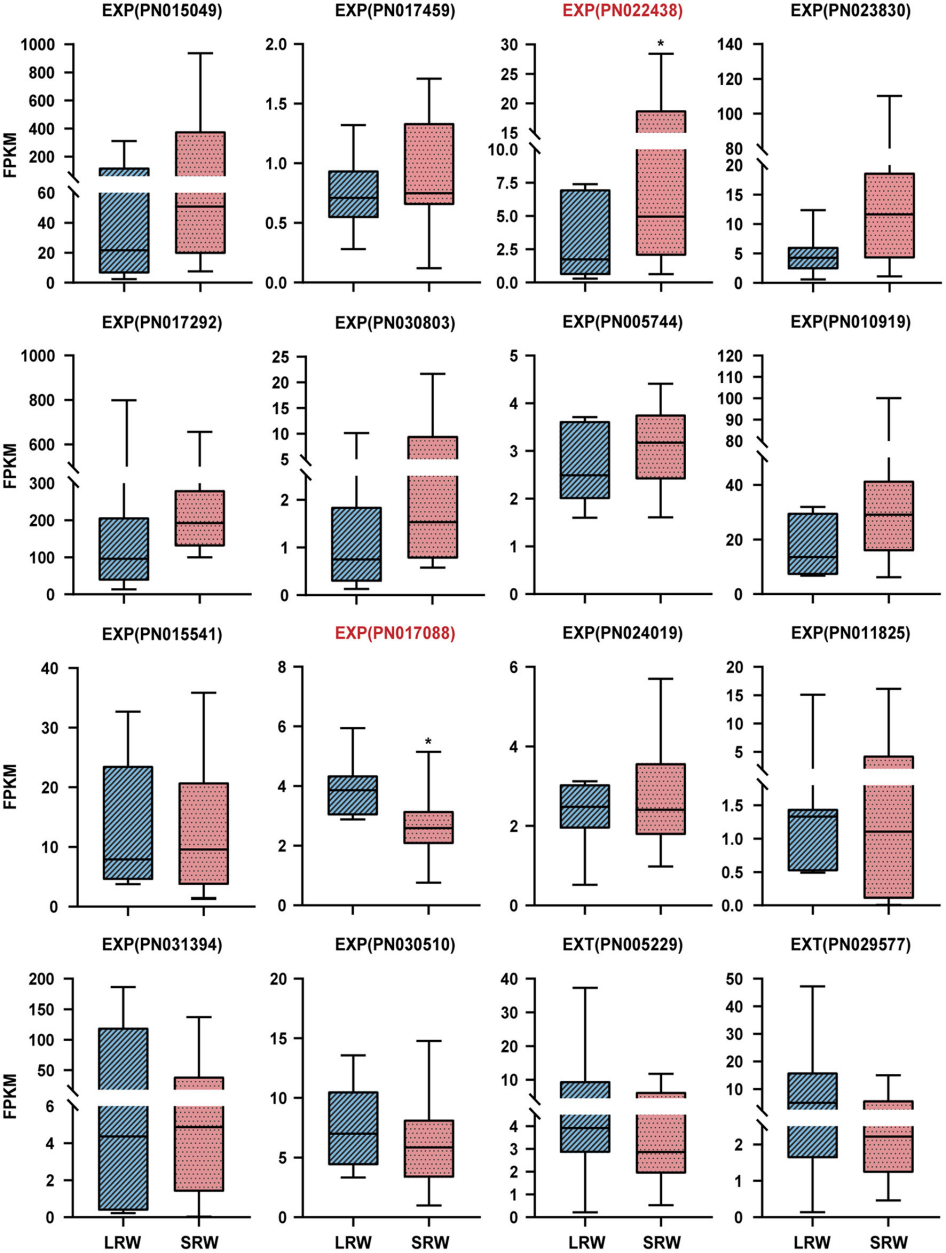
Expression pattern of *EXP*s and *EXT*s in LRW and SRW samples. EXPA, alpha-expansin; EXPB, beta-expansin; EXT, extension. Asterisks denote student's *t-*test significance: *P < 0.05.

Expansin (*EXP*) and extension (*EXT*), which possess a membrane-binding mode of GPI-anchored, could be associated with thickening of *P. notoginseng* root through cell wall expansion pathway (Li et al., 2019b; Zhou, 2019). *PnEXPA5* (PN022438) showed a prominently higher transcriptional level in SRW samples, while *PnEXPA4* (PN017088) expressed significantly higher transcriptional level in LRW samples, which was 1.44-fold higher than that in SRW samples (Figure 3). Phylogenetic tree using 39 *AtEXP*s and multiple sequence alignments indicated that *PnEXPA4* (PN017088) shows most similarity with *AtEXPA4*, and *PnEXPA5* (PN022438) is homologous with *AtEXPA5*, both possessing a DPBB domain and a pollen allergen domain. In addition, a WCNP domain in front of a HFD motif was found in PN017088, which is particularly owned in alpha-expansin (Supplementary Figures 1A,B). Since *AtEXPA4* had positively correlated with the cell wall thickness and root size (Ren et al., 2019), we speculated that *PnEXPA4* (PN017088) is associated with root enlargement of *P. notoginseng* as an essential factor.

### Higher Root Weight Was Accompanied by Higher Content of Ginsenoside Rb_1_ and Lower Content of Dencichine

**Figure 4:**
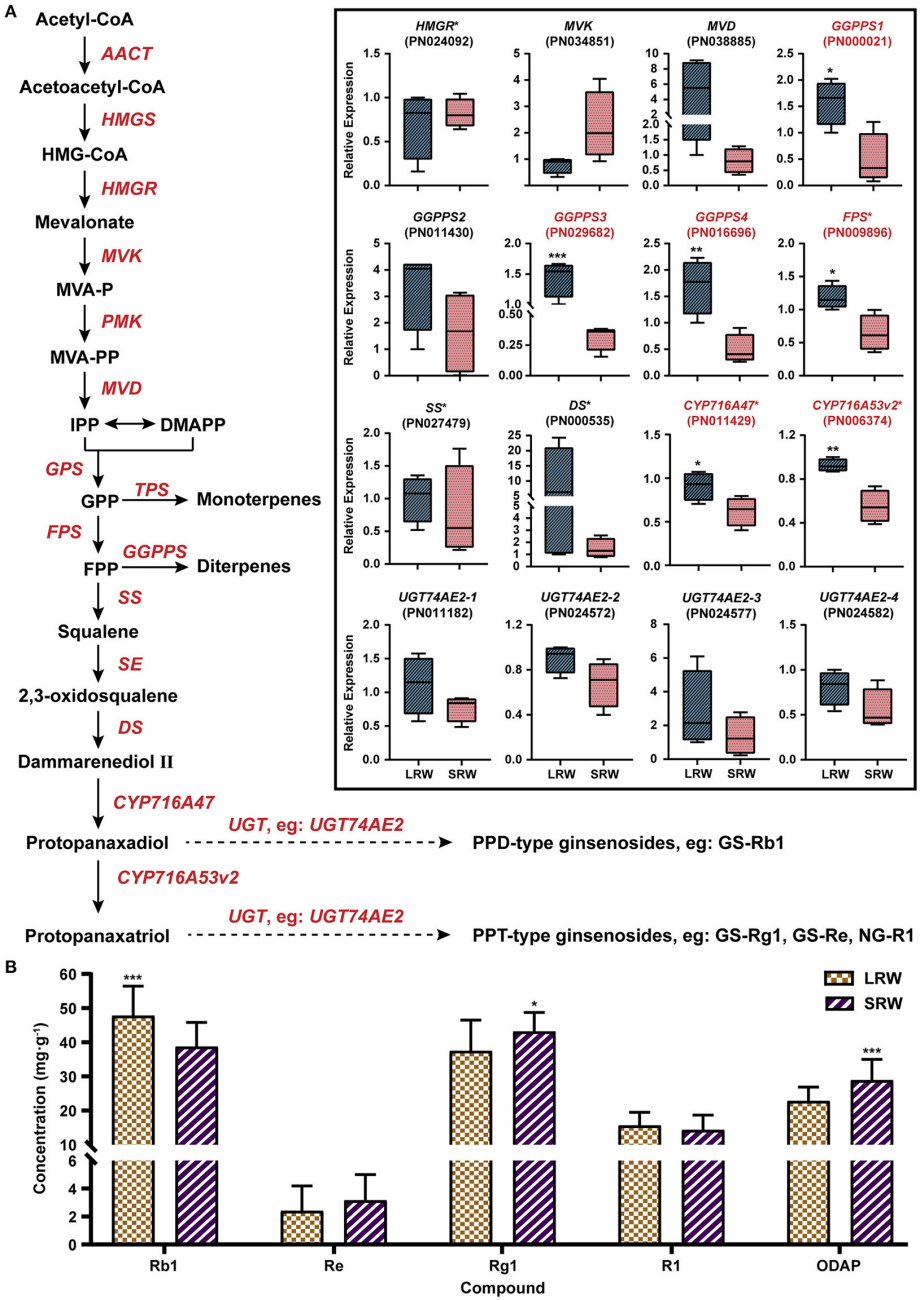
Expression pattern of genes related to biosynthesis of ginsenosides and content of active components. **(A)** Expression pattern of genes related to biosynthesis of ginsenosides in LRW and SRW groups. **(B)** Content of active components in LRW and SRW groups. AACT, acetyl-CoA C-acetyltransferase; HMGS, hydroxymethylglutaryl-CoA synthase; HMG-CoA, 3-hydroxy-3-methylglutaryl CoA; HMGR, hydroxymethylglutaryl-CoA reductase; MVK, mevalonate kinase; MVAP, mevalonate-5-phosphate; PMK, phosphomevalonate kinase; MVAPP, mevalonate-5-pyrophosphate; MVD, diphosphomevalonate decarboxylase; IPP, isopentenyl diphosphate; DMAPP, dimethylallyl diphosphate; GPS, geranyl pyrophosphate synthase; GPP, geranyl pyrophosphate; FPS, farnesyl diphosphate synthase; FPP, farnesyl diphosphate; GGPPS, geranylgeranyl pyrophosphate synthase; SS, squalene synthase; SE, squalene epoxidase; DS, dammarenediol-II synthase; CYP, cytochrome P450 proteins; UGT, UDP-glycosyltransferase; GS, ginsenoside; NG, notoginsenoside. Genes obtained * on upper right were reported to be functional. Genes in red tag had statistical significance. Asterisks denote Student's *t*-test significance: *P < 0.05, **P < 0.01, and ***P < 0.001.

To investigate whether the chemical composition is related to root size of *P. notoginseng*, we analyzed the expression of the genes related to ginsenosides biosynthesis, and the accumulation of ginsenosides and dencichine in LRW and SRW samples. In LRW, the expression level of *GGPPS1* (PN000021), *GGPPS3* (PN029682), *GGPPS4* (PN016696), *FPS* (PN009896), *CYP716A47* (PN011429), and *CYP716A53v2* (PN006374) was 3.25-, 4.54-, 3.42-, 1.84-, 1.46-, and 1.68-fold higher than that in SRW, respectively (Figure 4A). The content of ginsenoside Rb_1_ was significantly lower in SRW samples than that in LRW samples, while the content of dencichine in SRW samples was 1.28-fold higher than that in LRW samples (Figure 4B). It was reported that the content of protopanaxadiol and ginsenoside Rb_1_ was considerably increased under overexpression of *FPS* and *CYP716A47*, respectively (Han et al., 2012; Yang et al., 2017; Li et al., 2019a), indicating that higher expression level of *FPS* and *CYP716A47* led to remarkably higher content of ginsenoside Rb_1_ in LRW. In addition, WGCNA showed a significantly positive correlation between transcriptional level of *PnEXPA4* and *PnGGPPS*3, which were positively associated with root weight (Supplementary Figures 2A,B), suggesting that LRW samples with the thicker cell wall may be related to a higher content of ginsenosides.

### PnPHL8 Had Potential to Bind With *PnGAP, PnCYP716A47*, and *PnGGPPS3 in Vitro*

PHR transcription factor, belonging to MYB-CC family, participates in plant transcriptional responses to phosphate starvation (Wang et al., 2018b; Sega and Pacak, 2019). A PHR-like transcriptional factor PN024679 was filtrated through transcriptome analysis, which had a higher transcriptional level in SRW samples. PN024679 contains a 1074bp open reading frame (ORF) encoding 357 amino acids. A constructed phylogenetic tree using 14 AtPHLs indicated that PN024679 (named as PnPHL8) is homologous with AtPHL8 (Supplementary Figure 3A). Multiple sequence alignment showed PN024679 possessing a MYB DNA-binding domain and coiled-coil domain (Supplementary Figure 3B).

**Figure 5:**
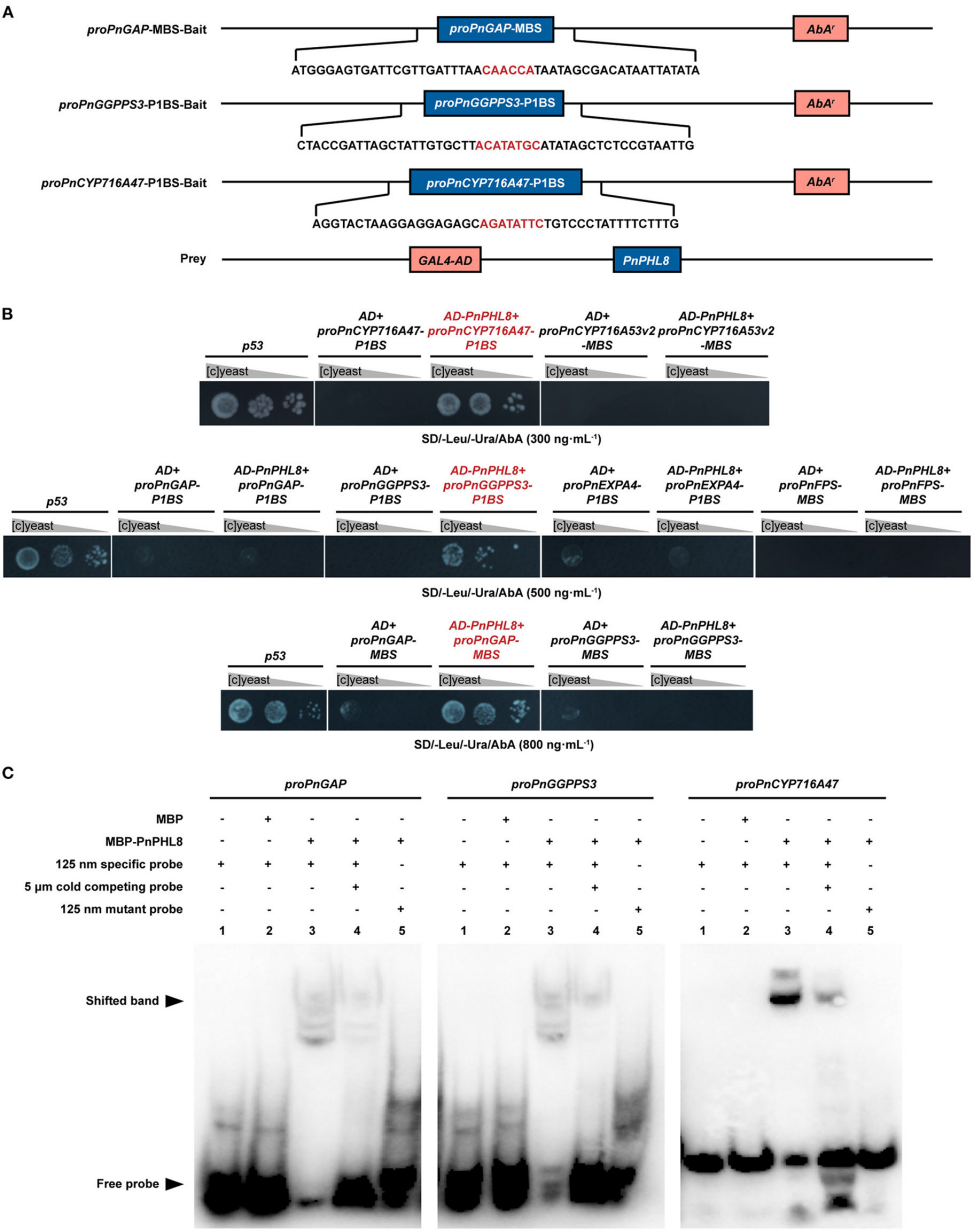
PnPHL8 binds with the promoter sequence of *PnGAP, PnCYP716A47*, and *PnGGPPS3*. **(A)** Structural schematics of baits and prey in yeast one-hybrid (Y1H) assay. **(B)** Y1H assay between PnPHL8 and PIBS domains as well as MBS domains of *proPnGAP* (promoter of *PnGAP*), *proPnEXPA4* (promoter of *PnEXPA4*), *proPnGGPPS3* (promoter of *PnGGPPS3*), *proPnCYP716A47* (promoter of *PnCYP716A47*), *proPnCYP716A53v2* (promotor of *PnCYP716A53v2*), and *proPnFPS* (promotor of *PnFPS*). Gray triangles represent dilution factor of the yeast concentration, while *p53* served as a positive control. **(C)** EMSA among PnPHL8, *proPnGAP, proPnCYP716A47*, and *proPnGGPPS3*. GAP, glycosylphosphatidylinositol-anchored protein; PHL, phosphate starvation response transcription factor like; AbA, aureobasidin.

We investigated whether PnPHL8 regulates genes related to root size and ginsenosides biosynthesis *in vitro* by Y1H method. Promoter sequences of *PnGAP, PnEXPA4, PnGGPPS3, PnFPS, PnCYP716A47*, and *PnCYP716A53v2* were PCR-amplified, all of which except *proPnFPS* and *proPnCYP716A53v2* contained either a P1BS binding site (GNATATNC) or a P1BS-like element (Sun, 2015). MBS domain was also existed in *proPnGAP, proPnFPS, proPnCYP716A53v2*, and *proPnGGPPS3* (Ding et al., 2017; Mabuchi et al., 2018). Specific P1BS domain and MBS domain of *proPnGAP, proPnEXPA4, proPnGGPPS3, PnFPS, PnCYP716A47*, and *PnCYP716A53v2* were integrated into yeast, individually. After cotransformation with pGADT7-PnPHL8, we found that yeast strains carrying proPnGAP-MBS, proPnCYP716A47-P1BS, and proPnGGPPS3-P1BS could grow on SD minus leucine and uracil with aureobasidin (Figures 5A,B).

To avoid false-positive results caused by Y1H assay, we further performed EMSA to verify the interaction of PnPHL8 with the promoters of *PnGAP, PnCYP716A47*, and *PnGGPPS3 in vitro*. Fragments of *proPnGAP, PnCYP716A47*, and *proPnGGPPS3*, containing MBS or P1BS motif, were synthesized as specific probes for EMSA, while mutated probes were synthesized by replacing MBS/P1BS motif with poly-A/T. We found that MBP-PnPHL8 was able to bind to the promoter fragments of *PnGAP, PnCYP716A47*, and *PnGGPPS3*, but failed to bind to the mutated probes. Moreover, the cold competing probes, which contain high concentration of unlabeled promotor fragments, impaired the interaction between PnPHL8 and specific probes (Figure 5C). These results indicate that PnPHL8 specifically binds the promoters of *PnGAP, PnCYP716A47*, and *PnGGPPS3 in vitro*, suggesting that PnPHL8 may synergistically regulate biosynthesis of diterpenoid and triterpenoid, as well as cell wall architecture.

## Discussion

### Cell Wall Architecture Involved in Transition of Root Size of *P. notoginseng*

In this study, we revealed that the root of *P. notoginseng* exhibited a faster growth rate and better-developed root system during the past 100 years. Plant possessing larger diameter of vessels usually has a faster growth rate and water carrying capacity of vessel (Ian et al., 2008). Lignin also serves as a major component of vessel, which agreed with the significantly larger vessels and a higher content of lignin in LRW samples. In LRW samples, width of root, thickness of vessels and xylem cell wall, and the content of cellulose and callose were significantly higher than that in SRW samples, which might be due to the higher expression of *PnGAP* and *PnEXPA4* in LRW. It was reported that GAPs related to cell wall architecture were mainly expressed in xylem vessels and adjacent parenchyma cells and then functioned in secondary wall deposition (Loopstra and No, 2000; Dahiya et al., 2006; Dai, 2015). In addition, the expression of *EXPA4* and *GAP*s was positively related to the cell wall thickness, with *GAP*s also showing a positive correlation with the content of cellulose (Sun, 2013; Ben-Tov et al., 2015; Mcnair, 2015; Ren et al., 2019).

*P. notoginseng* is now usually cultivated at higher altitude, lower light transmittance, lower temperature, and lower precipitation condition, compared with earlier times. Lower temperature increased the gene expression level of *PnGAP* (Daisuke et al., 2016; Zhao et al., 2020), while cold acclimation, water stress, and phosphorus application led to higher expression of *EXPA* and *EXT* (Bian, 2006; Kozbial et al., 2010; Li et al., 2013; Han et al., 2014). The expression of *EXPA*s was upregulated under green shade within limits, but was strongly inhibited under dark treatment (Ping et al., 1994; Sasidharan et al., 2009; Liu et al., 2011). The vessel diameter and vessel wall thickness showed a significant increase under drought (Xu and Chen, 2012; Aref et al., 2013). Following the transition of cultivation measures, lower temperature and drought could increase the vessel diameter and vessel wall thickness, and a faster growth rate and then a bigger root size of *P. notoginseng* were formed. Higher expression of *PnGAP* in vessels and xylem parenchyma contributed to a higher content of cellulose and callose, while higher expression of *PnEXPA4* led to better-architected cell wall. In addition, cell wall-mediated resistance is an important part of plant immune response system, to which the cell wall thickness was positively related (Aquije et al., 2010; Rachid et al., 2016), and a large root size was reportedly associated with resistance (Chloupek, 2010). The PHR transcription factor family participates in plant transcriptional responses to phosphate starvation (Wang et al., 2018b; Sega and Pacak, 2019). Lower application of phosphate in *Qing* dynasty might result in higher transcriptional level of *PnPHL8*, which subsequently influenced the expression of *PnGAP* and affected cell wall architecture and expansion of *P. notoginseng*. Based on this, we speculate that cell wall architecture played an important role in transition of root size from *Qing* dynasty to the present.

### The Variation of Chemical Components Content and Root Size of *P. notoginseng* Might Lead to Transition in Clinical Usage

Ginsenoside Rb_1_ and dencichine, the major compounds in root of *P. notoginseng*, show diverse pharmacological activities. Ginsenoside Rb_1_ has great effects on vascular endothelial function improvement (Ohashi et al., 2006), cerebral ischemia protection (Yuan et al., 2007), myocardial preservation (Zhao et al., 2010), and neuroprotection (Jin et al., 2005; Liang et al., 2010), while dencichine was used for the treatment of injury induced trauma, and its hemostatic function was proven by clinical practice (Zhang and Yu, 2010). Modern cultivation condition, for example, lower temperature and drought, is beneficial to the accumulation of saponins in *P. notoginseng* (Konsler et al., 1990; Yu et al., 2001; Liu et al., 2016b; Ma et al., 2021). Here, we also found high content of ginsenosides Rb_1_ in LRW samples. In addition, the PHL transcription factor negatively regulates secondary metabolism such as carotenoid (Lu et al., 2021), indicating that the higher expression of *PnPHL8* in SRW samples may lead to a lower content of secondary metabolism such as ginsenoside Rb_1_. In contrast, the content of dencichine was lower in LRW samples, which may be resulted from the decreased activity of SAT induced by drought (Ahmad et al., 2016; Yang et al., 2021). As recorded in herbal records, *P. notoginseng* in *Qing* dynasty was mostly applied externally or prepared into powder for hemostasis (Chen, 2009). However, prescription containing *P. notoginseng* with a higher content of ginsenoside Rb_1_ in recent years was mainly used to treat heart diseases and injuries (Chen et al., 2017). The change in root size, ginsenoside Rb_1_ and dencichine content of *P. notoginseng* from *Qing* dynasty to modern cultivation era, may also influence the clinical usage.

Triterpenoid is reported as a regulator of cell wall biosynthesis (Jozwiak et al., 2020), and ginsenoside Rb_1_ was localized to degrading primary cell wall of xylem in root of *Panax ginseng* (Yokota et al., 2011). In addition, a significantly positive correlation between the expression of *PnEXPA4* and *PnGGPPS*3 was also observed by WGCNA, suggesting that cell wall architecture pathway and ginsenosides biosynthesis pathway may jointly participate in root enlargement of *P. notoginseng* during past 100 years.

## Conclusion

**Figure 6:**
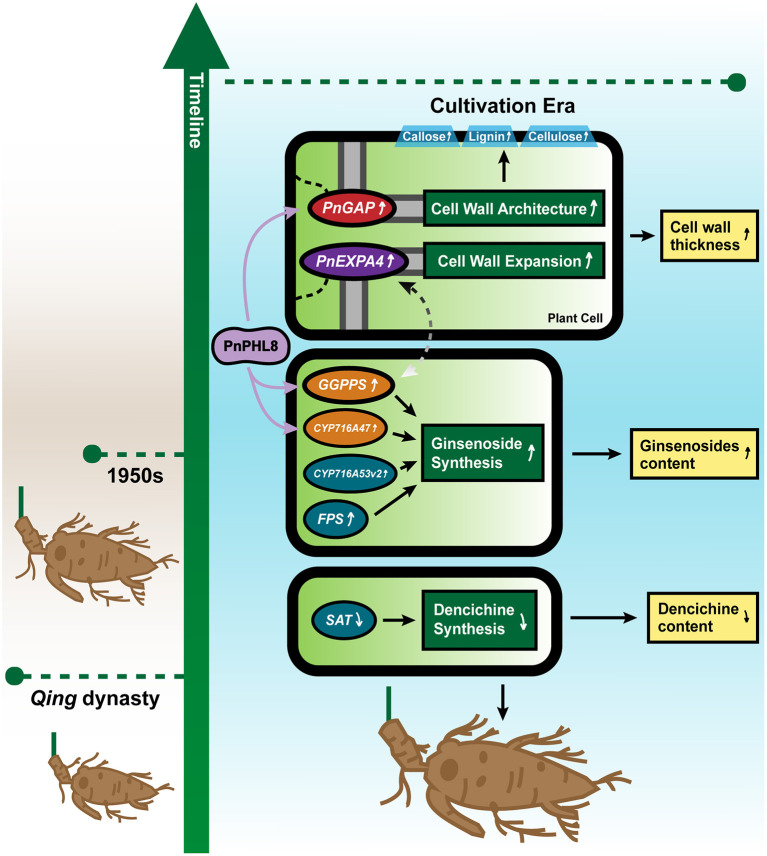
Probable molecular mechanism model for cultivation influencing root architecture and chemical component of *P. notoginseng*. Changes in root size and active component accumulation of *P. notoginseng* are influenced by related genes and affected by changes in the cultivation environment. Increased expression of *PnGAP* and *PnEXPA4* results in the thicker cell wall and a higher content of lignin, cellulose, and callose in *P. notoginseng* of cultivation era. Higher expression of *GGPPS, FPS, CYP716A47*, and *CYP716A53v2* contributes to a higher content of ginsenosides, while lower expression of *SAT* might result in a lower content of dencichine. A PnPHL8 transcriptional factor participates in transcriptional regulation of *PnGAP, PnCYP716A47*, and *PnGGPPS3*, while dotted line between *PnEXPA4* and *PnGGPPS3* indicates that a probable positive correlation may exist.

From *Qing* dynasty to modern times, cultivation increased the root size and changed the content of ginsenoside and dencichine of *P. notoginseng*. In this study, we revealed that large root size of modern *P. notoginseng* should be due to the high expression of *PnGAP* and *PnEXPA4*, by promoting better-architected cell walls and larger vessels. *GGPPS, FPS, CYP716A47*, and *CYP716A53v2* involved in ginsenosides biosynthesis pathway are also induced to contribute to a relatively higher content of ginsenosides, while depressed expression of *SAT* in LRW sample affected dencichine biosynthesis, leading to transition toward clinical efficacy from *Qing* dynasty to cultivation era. PnPHL8 participates in transcriptional regulation of *PnGAP, PnCYP716A47*, and *PnGGPPS3*, modulating cell wall architecture and ginsenosides biosynthesis pathway (Figure 6). Our results toward *P. notoginseng* of 2 eras separated by 100 years provided enlightenment on how long cultivation affected root size, chemical composition, and clinical usage.

## Data Availability Statement

The 10 *P. notoginseng* RNA-seq profiles (r9, r14, r20, r28, r29, r35, r36, r45, r48 and r55) are available in National Genomics Data Center under the GSA accession number CRA006118. The remaining 11 *P. notoginseng* RNA-seq profiles (r1, r13, r15, r18, r19, r23, r26, r32, r33, r53 and r59) have been deposited at DDBJ/EMBL/GenBank under the accession GFRX00000000 (Zheng et al., 2017).

## Author Contributions

X-MC, L-QH, and YY designed the study. M-YY, Z-YH, P-RL, HZ, and YJ performed the experiments. M-YY, Z-YH, and H-SP analyzed data. M-YY, Z-YH, and YY wrote the manuscript. All authors discussed the results and commented on the manuscript.

## Funding

This research was financially supported by the National Natural Science Foundation of China (NSFC) (81891013/81891010), the Scientific and Technological Innovation project of China Academy of Chinese Medical Science (C12021A041), and the Key project at the central government level for the ability to the establishment of sustainable use for valuable Chinese Medicine Resources (2060302).

## Conflict of Interest

The authors declare that the research was conducted in the absence of any commercial or financial relationships that could be construed as a potential conflict of interest.

## Publisher's Note

All claims expressed in this article are solely those of the authors and do not necessarily represent those of their affiliated organizations, or those of the publisher, the editors and the reviewers. Any product that may be evaluated in this article, or claim that may be made by its manufacturer, is not guaranteed or endorsed by the publisher.
