# Supplementary figures and images for: Increasing Expression of PnGAP and PnEXPA4 Provides Insights Into the Enlargement of Panax notoginseng Root Size From Qing Dynasty to Cultivation Era
Source: Front Plant Sci. 2022 May 20;13:878796. doi: 10.3389/fpls.2022.878796 (PMC9164015; doi:10.3389/fpls.2022.878796)

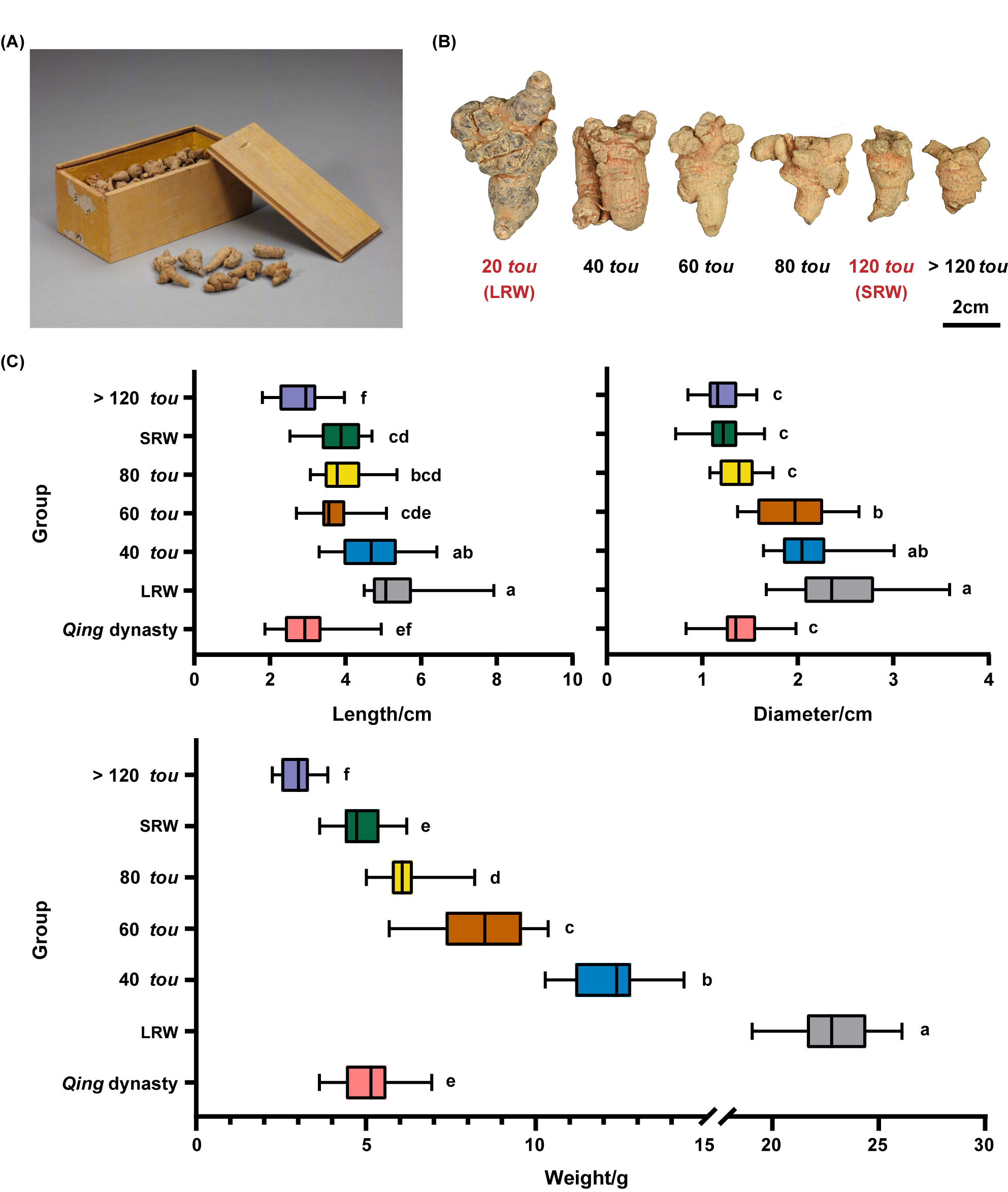

Supplement: Supplementary file 1 [file Data_Sheet_1.ZIP › Supplementary Material Presentation/Figure 1.tif]

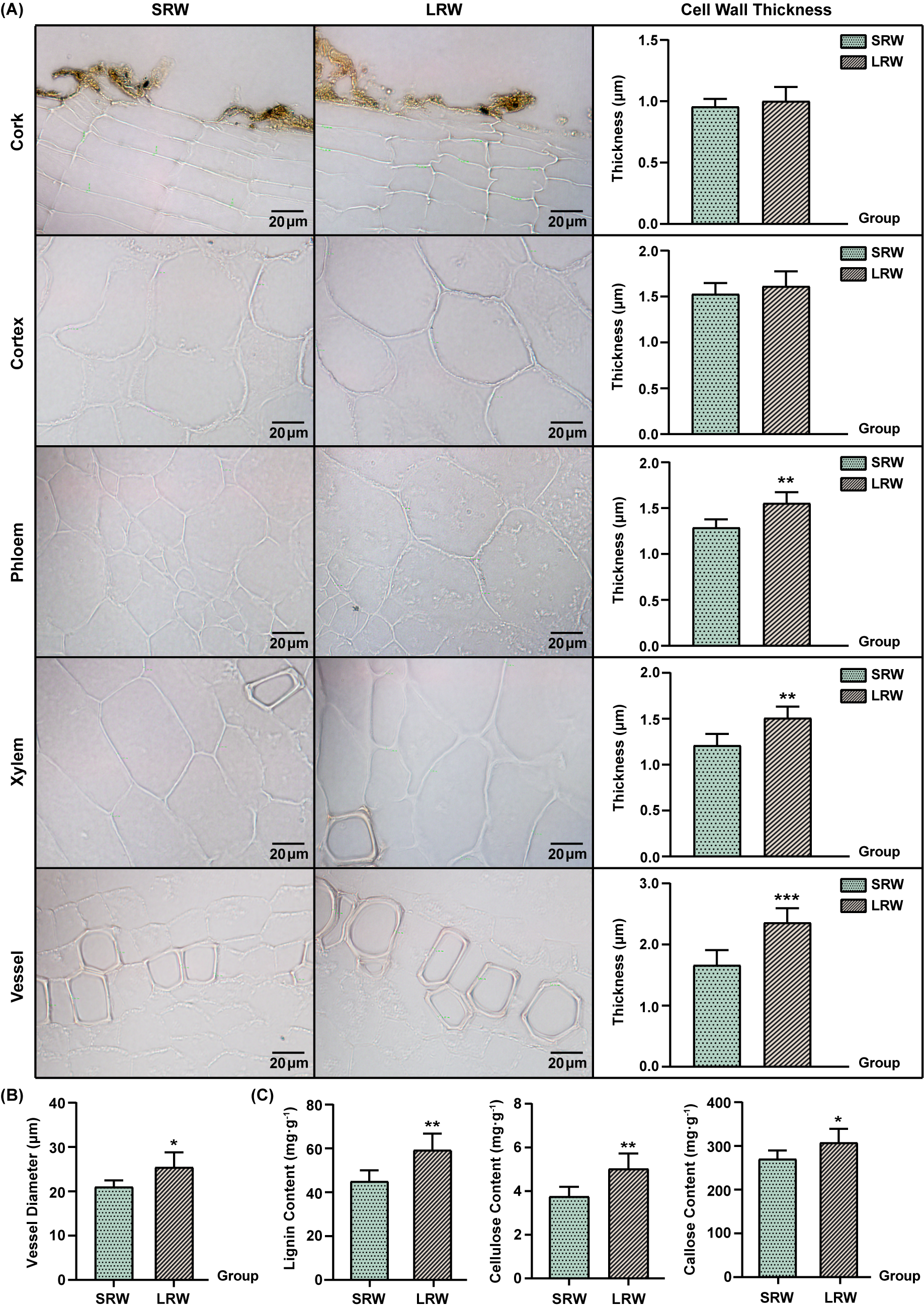

Supplement: Supplementary file 1 [file Data_Sheet_1.ZIP › Supplementary Material Presentation/Figure 2.tif]

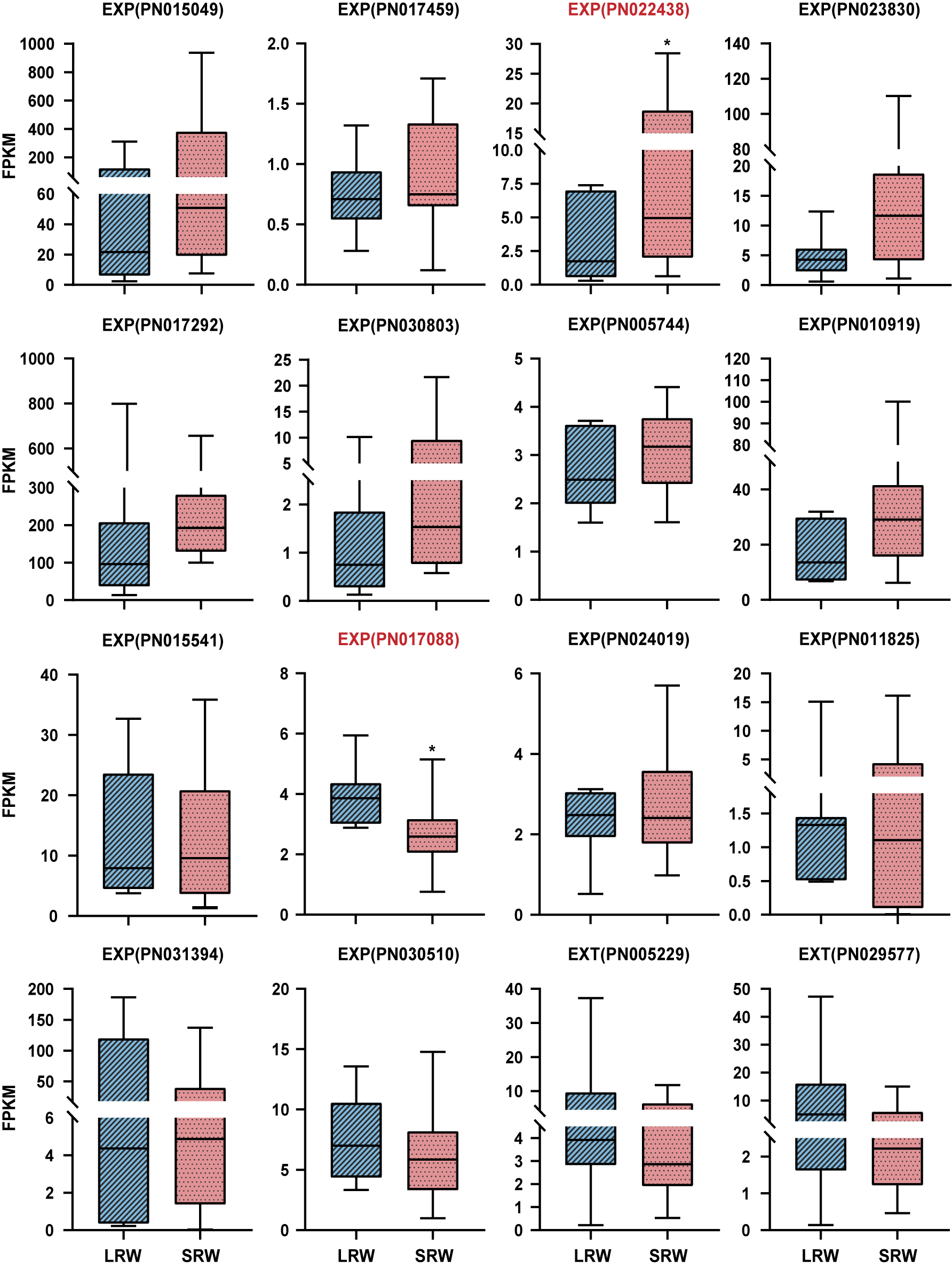

Supplement: Supplementary file 1 [file Data_Sheet_1.ZIP › Supplementary Material Presentation/Figure 3.tif]

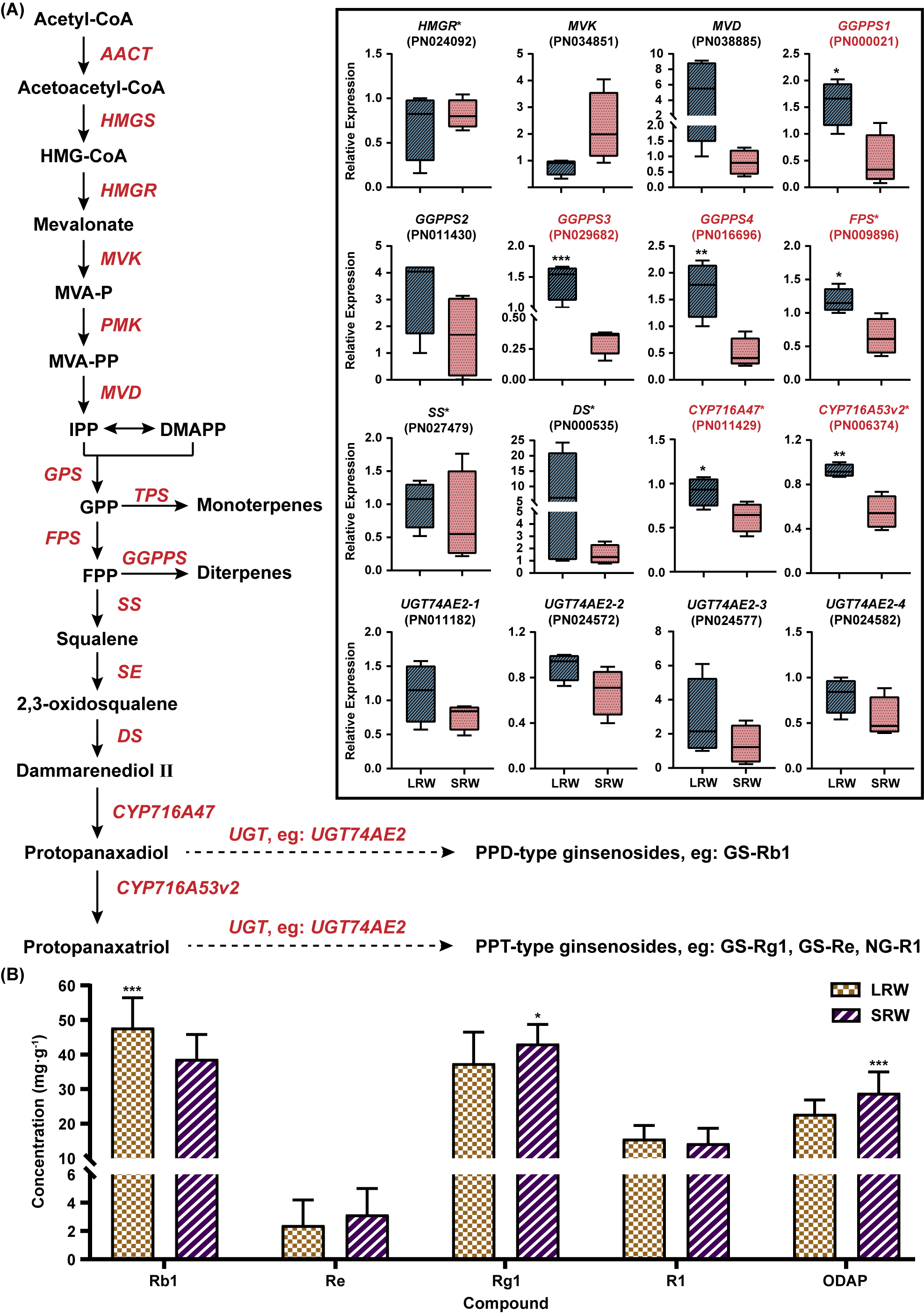

Supplement: Supplementary file 1 [file Data_Sheet_1.ZIP › Supplementary Material Presentation/Figure 4.tif]

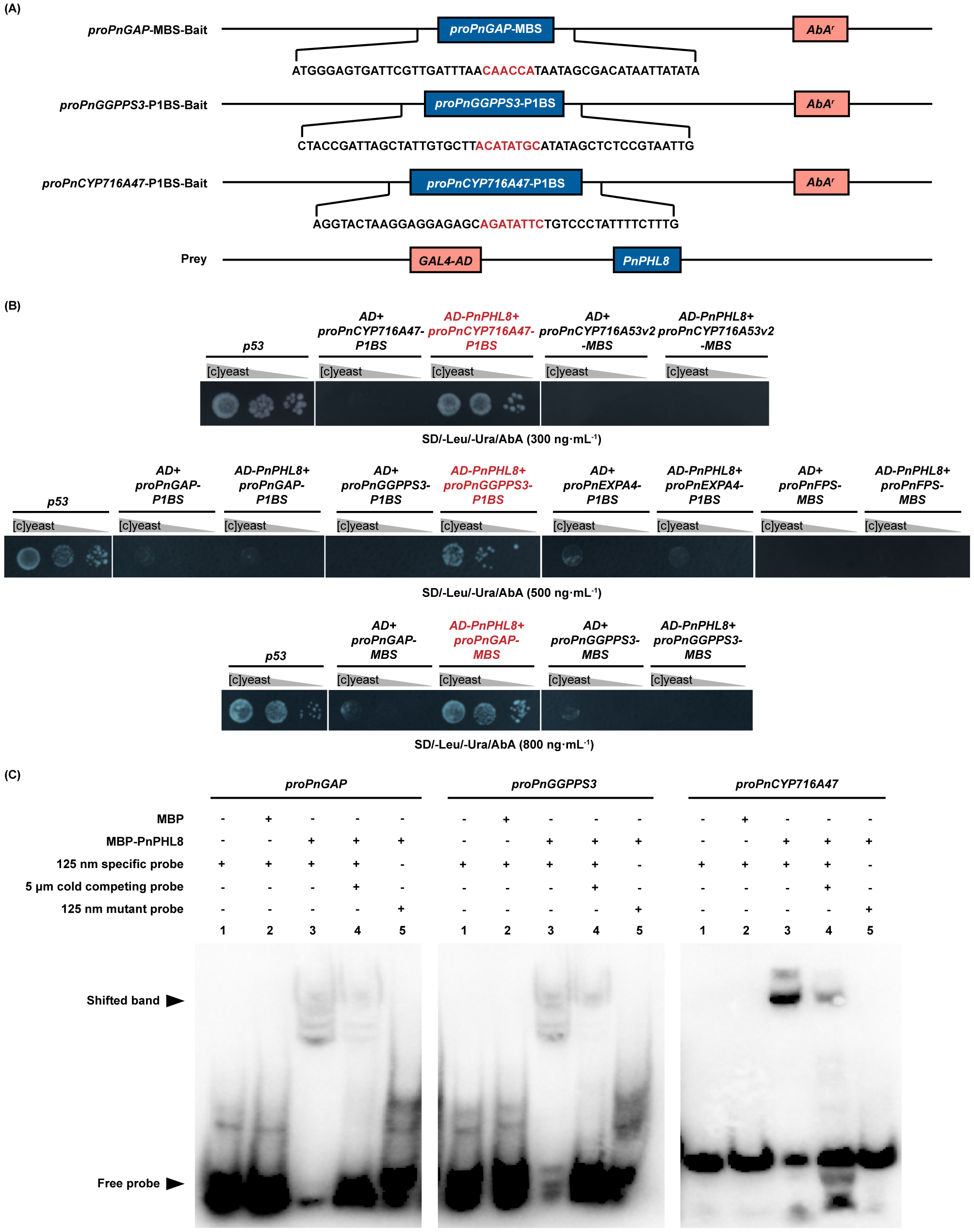

Supplement: Supplementary file 1 [file Data_Sheet_1.ZIP › Supplementary Material Presentation/Figure 5.tif]

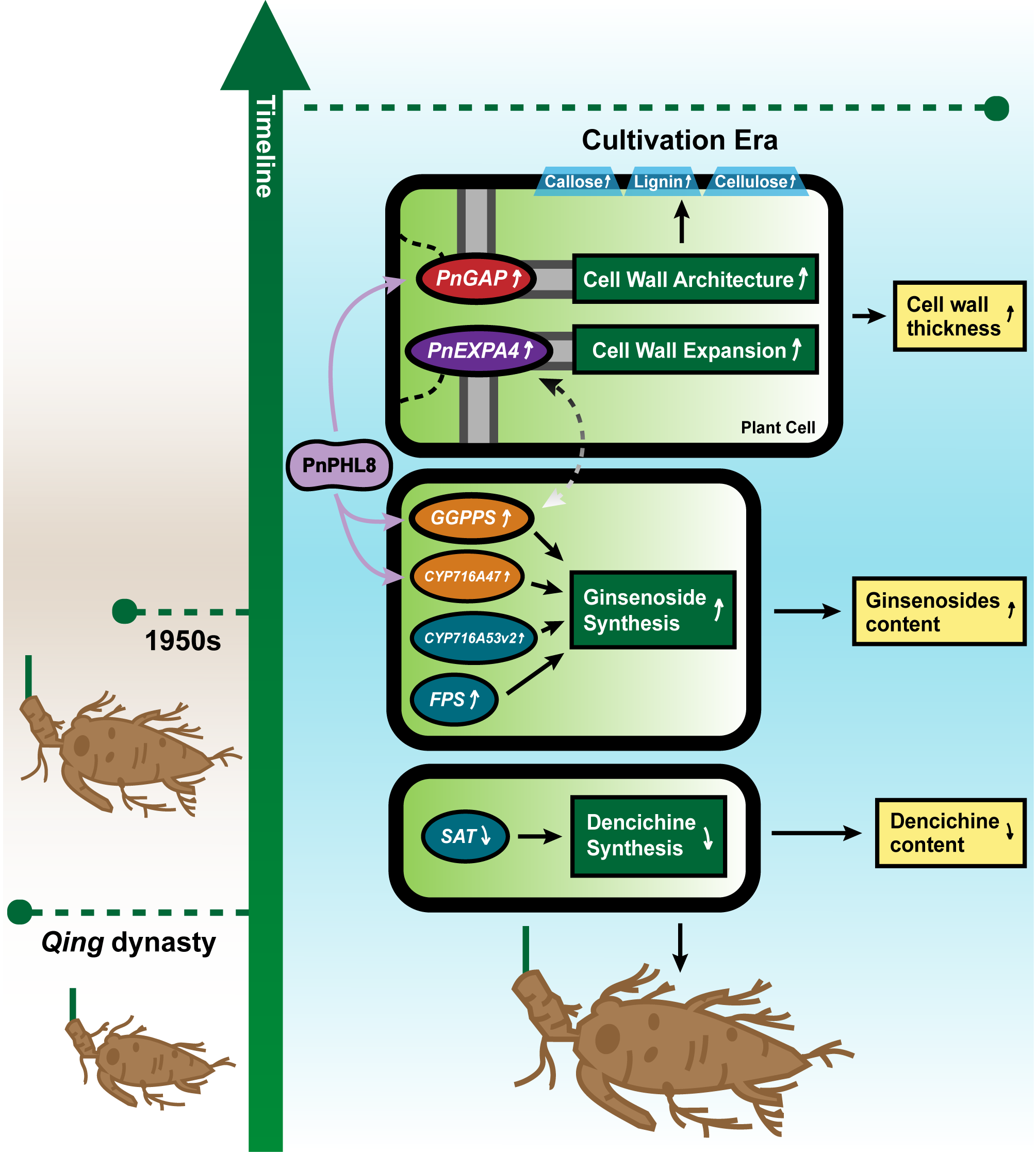

Supplement: Supplementary file 1 [file Data_Sheet_1.ZIP › Supplementary Material Presentation/Figure 6.tif]

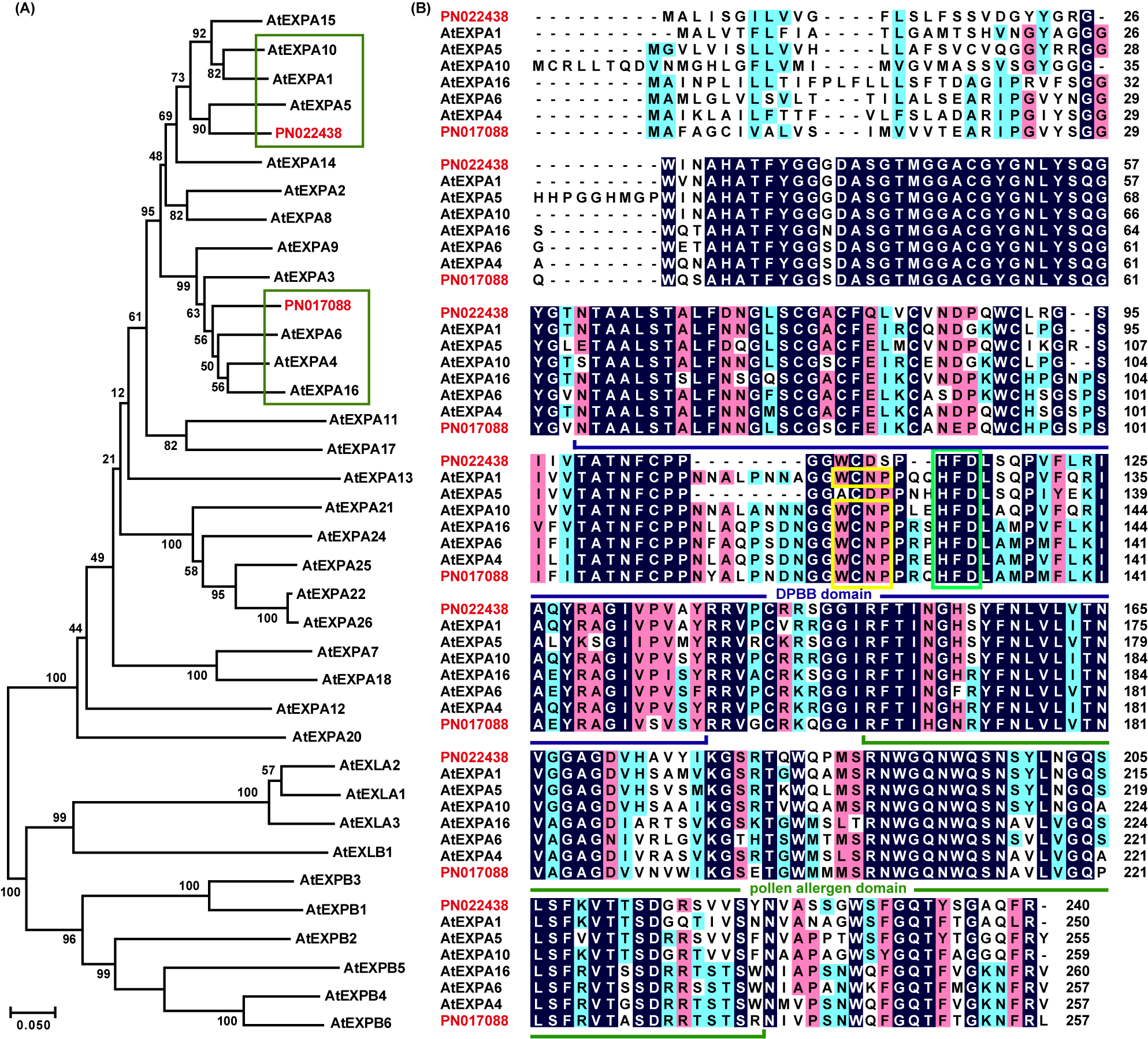

Supplement: Supplementary file 3 [file Image_1.TIF]

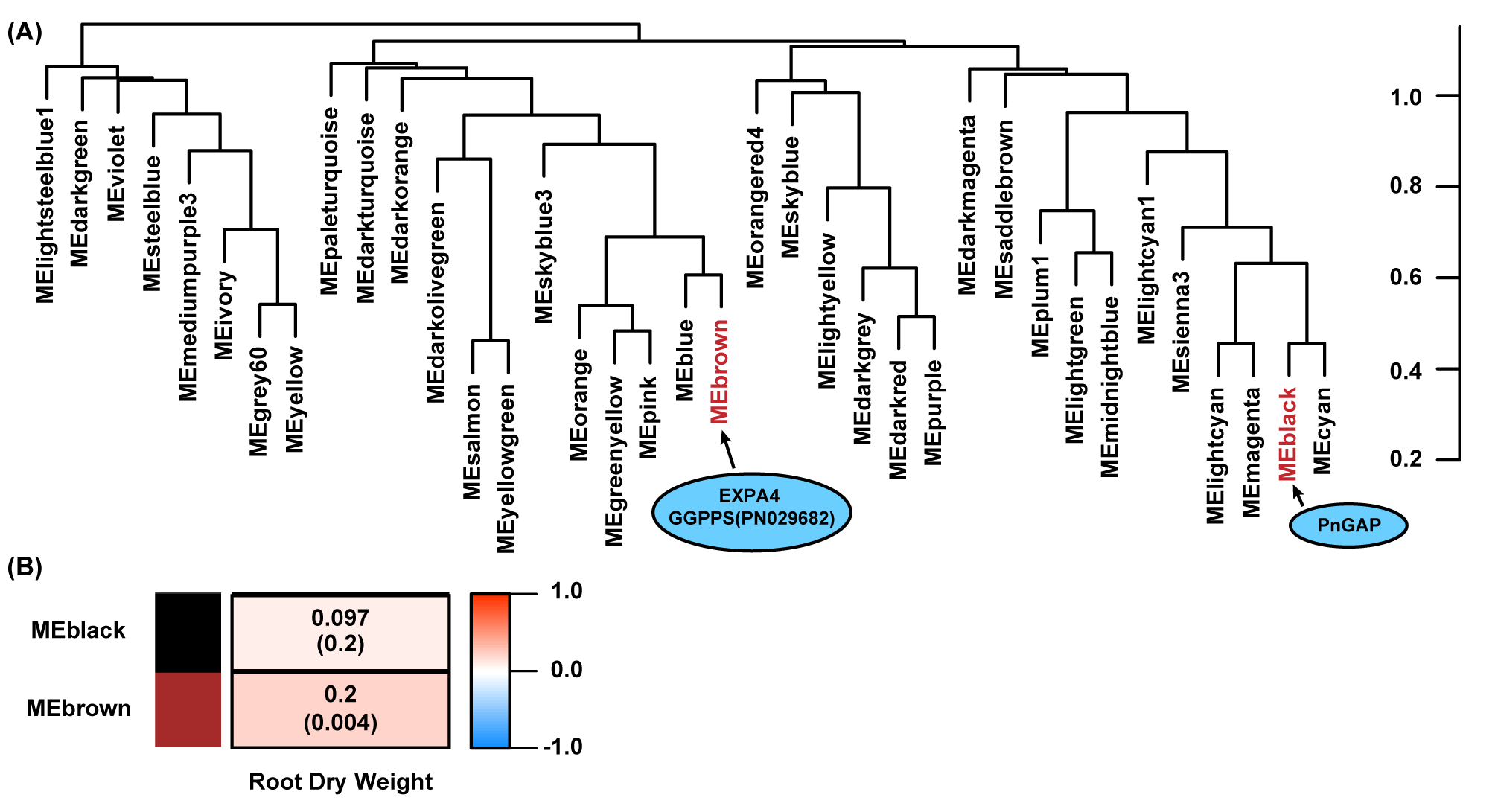

Supplement: Supplementary file 4 [file Image_2.TIF]

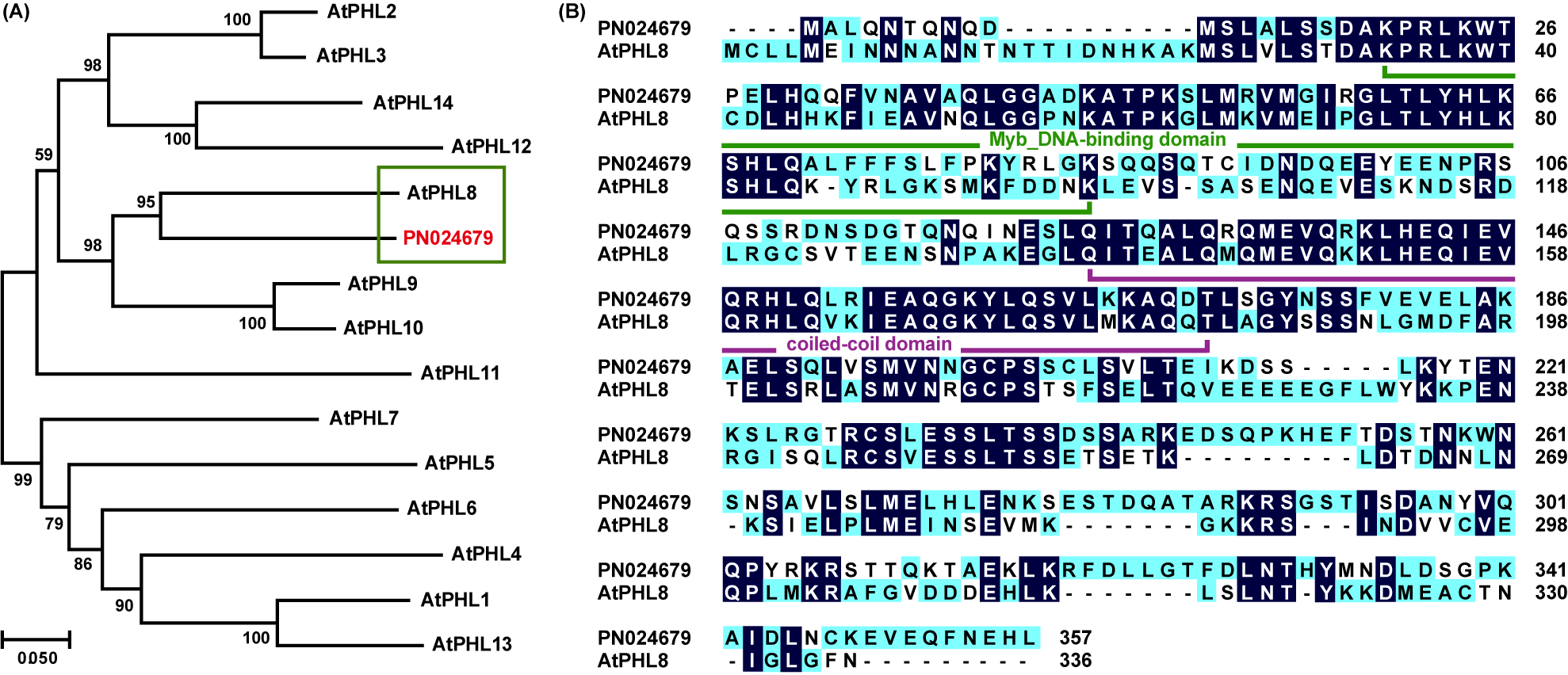

Supplement: Supplementary file 5 [file Image_3.TIF]
