# Supplementary material for: Increasing Expression of PnGAP and PnEXPA4 Provides Insights Into the Enlargement of Panax notoginseng Root Size From Qing Dynasty to Cultivation Era
Source: Front Plant Sci. 2022 May 20;13:878796. doi: 10.3389/fpls.2022.878796 (PMC9164015; doi:10.3389/fpls.2022.878796)
Supplement: Supplementary file 2 [file Data_Sheet_2.docx]

Supplementary Material

# Supplementary Figures and Tables

## Supplementary Figures


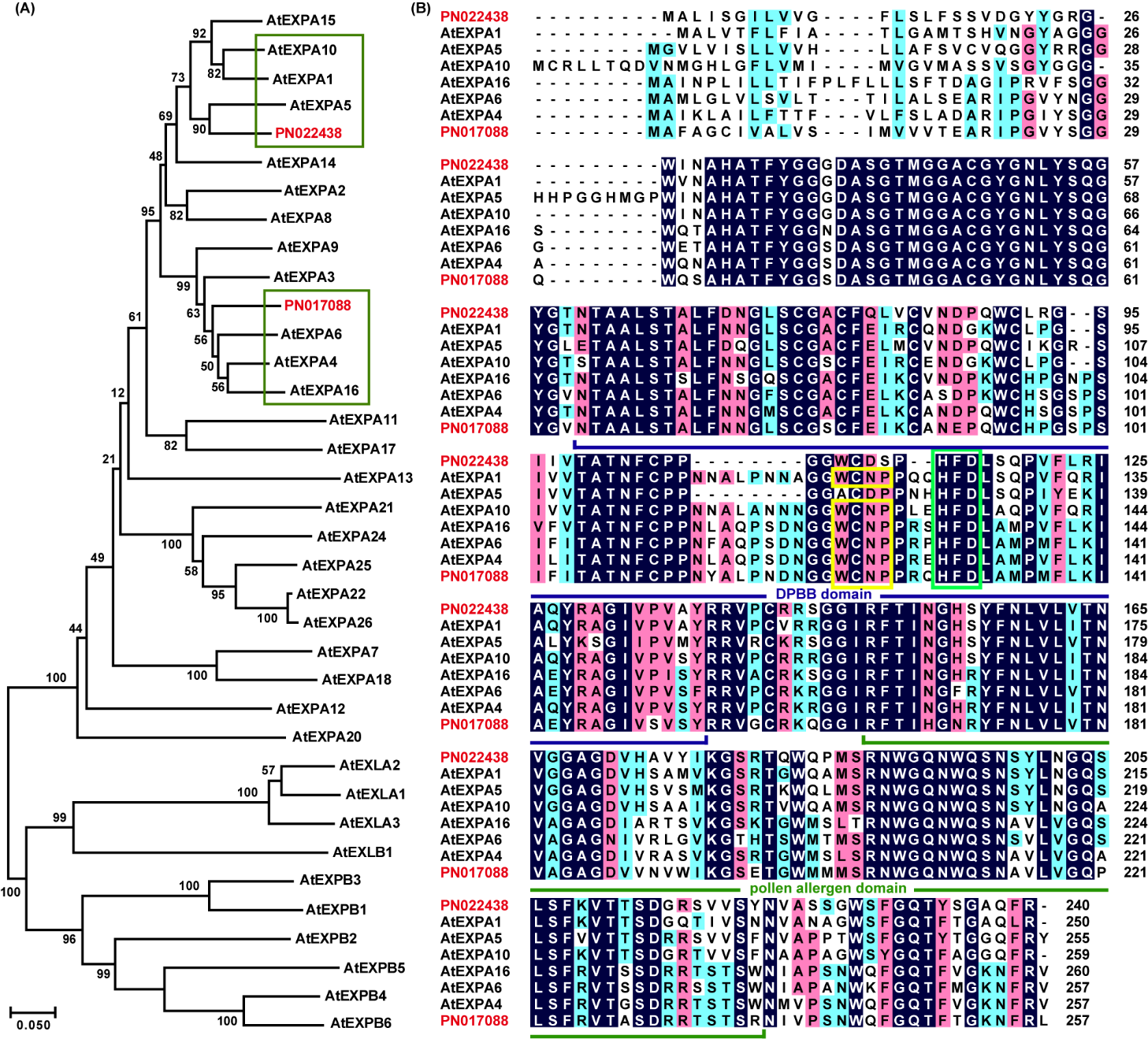


**Supplementary Figure 1.** **Phylogenetic tree and multiple sequence alignment of PN022438 and PN017088.** (A) Phylogenetic reconstruction of PN022438 and PN017088. Bootstrap values from 1000 replicates were used to assess the robustness of the Jones-Taylor-Thornton (JTT) neighbor-joining tree. (B) Multiple sequence alignment of PN022438 and PN017088. The yellow frame indicates a WCNP domain, while green frame represents a HFD motif.


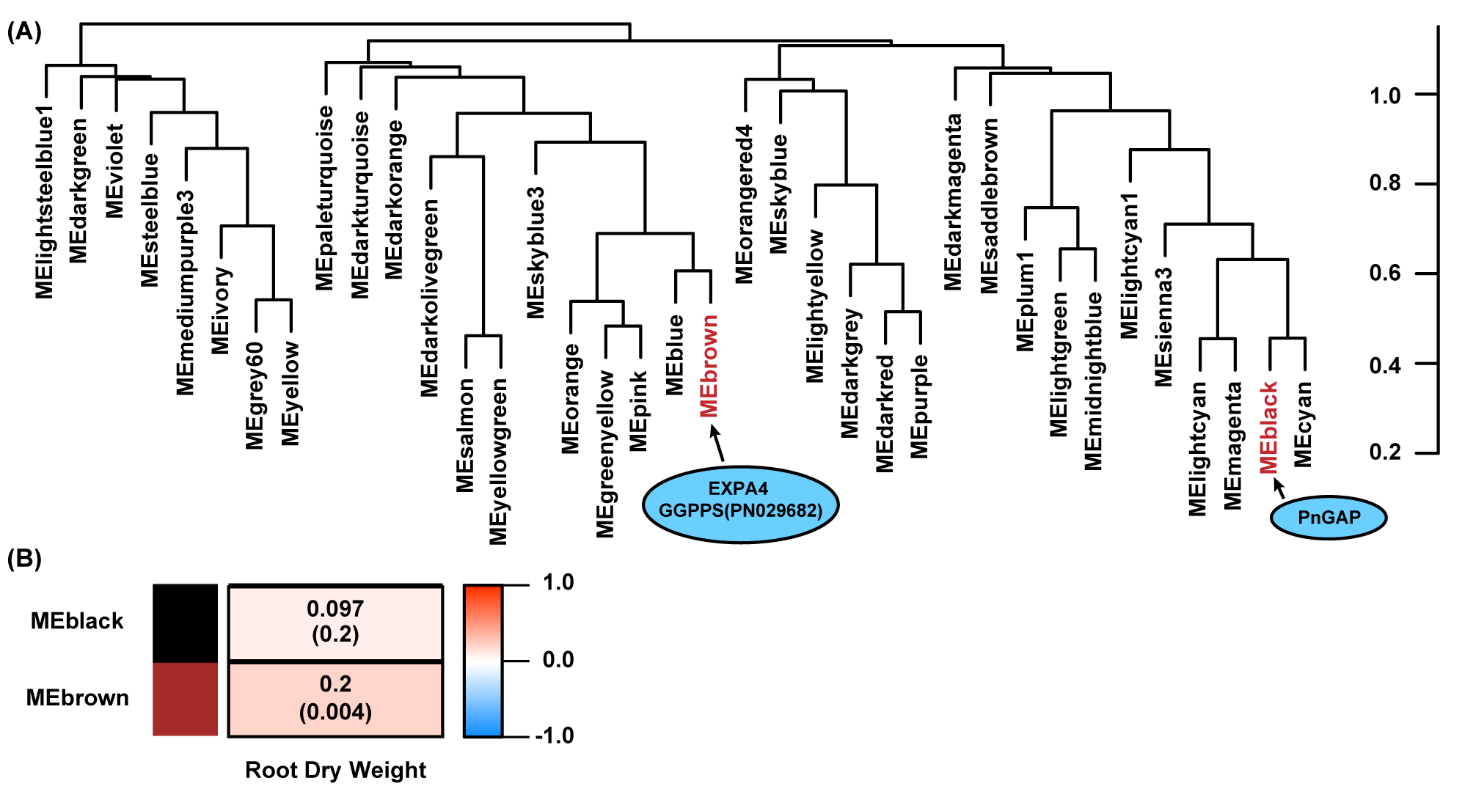


**Supplementary Figure 2. WGCNA module identification and correlation analysis.** (A) Clustering dendrograms of genes and module detecting. Each branch of gene clustering tree corresponds to one module. EXPA4 and GGPPS (PN029682) were both clustered into MEbrown module. PnGAP was clustered into MEblack module. (B) Heatmap of the correlation between modules and traits. Each row corresponds to a module, and each column represents a trait. The correlation coefficient and the corresponding p-value are shown in each cell. The red lattice corresponds to a positive relationship between agricultural traits with the module, while the blue lattice represents a negative correlation. The degree of color indicates degree of correlation.


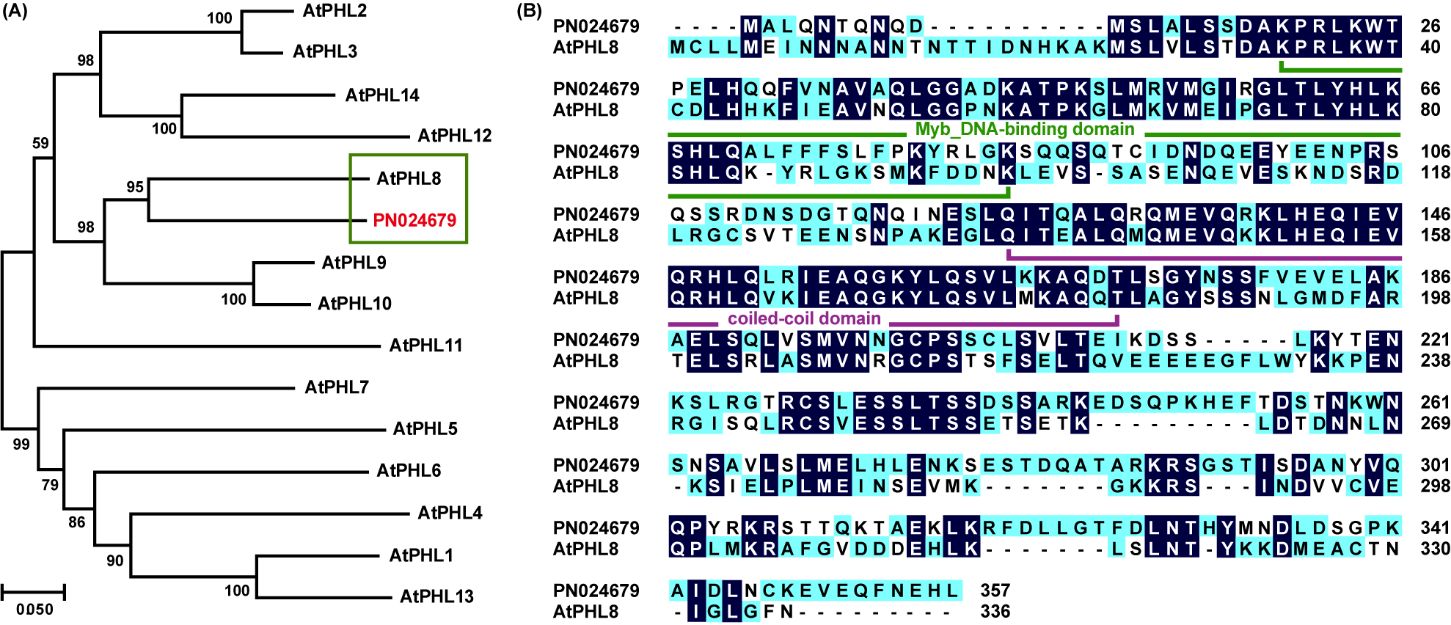


**Supplementary Figure 3. Phylogenetic tree and multiple sequence alignment of *PnPHL8*.** (A) Phylogenetic reconstruction of PN024679. Bootstrap values from 1000 replicates were used to assess the robustness of the Jones-Taylor-Thornton (JTT) neighbor-joining tree. (B) Multiple sequence alignment of PN024679.

## Supplementary Tables

**Supplementary Table 1. The characteristics of 21 *P. notoginseng* used in the study**

| No. | Root weight/g | Dry weight/g (Xu, 2016) | Group |
| --- | --- | --- | --- |
| r1 | 11.6 | 5.22 | SRW |
| r9 | 31.1 | 13.99 | LRW |
| r13 | 11.2 | 5.04 | SRW |
| r14 | 13.3 | 5.98 | SRW |
| r15 | 29.4 | 13.23 | LRW |
| r18 | 30.1 | 13.54 | LRW |
| r19 | 9.4 | 4.23 | SRW |
| r20 | 10.1 | 4.54 | SRW |
| r23 | 10.7 | 4.81 | SRW |
| r26 | 10.3 | 4.63 | SRW |
| r28 | 28.8 | 12.96 | LRW |
| r29 | 28.9 | 13.00 | LRW |
| r32 | 10.5 | 4.72 | SRW |
| r33 | 13.5 | 6.07 | SRW |
| r35 | 13.2 | 5.94 | SRW |
| r36 | 10.3 | 4.63 | SRW |
| r45 | 29.3 | 13.18 | LRW |
| r48 | 44.4 | 19.98 | LRW |
| r53 | 13.3 | 5.98 | SRW |
| r55 | 12.1 | 5.44 | SRW |
| r59 | 11.2 | 5.04 | SRW |

**Supplementary Table 2. Primers used in qRT-PCR, promotor PCR amplification and bait construction**

| Gene | Sequence (5’-3’) |
| --- | --- |
| *qRT-PCR primers* |  |
| 18s-q-F | AGCCTTGCGACCATACTCCC |
| 18s-q-R | CCATAAACGATGCCGACCAG |
| *HMGR*-024092-q-F: | GTACTGTTGGAGGTGGAACGCA |
| *HMGR*-024092-q-R: | CATGTGGCTCCTAACAAGCTGC |
| *MVK*-034851-q-F: | GTACTGTTGGAGGTGGAACGCA |
| *MVK*-034851-q-R: | CATGTGGCTCCTAACAAGCTGC |
| *MVD*-038885-q-F: | TCCCTCCACATTCCAACACTGAT |
| *MVD*-038885-q-R: | AGGAGAGCTCGACTATCAGGTAGCA |
| *GGPPS*-000021-q-F: | CAAAGTCTTCCCAAGAATTGGGA |
| *GGPPS*-000021-q-R: | TCCCTATAAGCAATGTAATTCGCC |
| *GGPPS*-011430-q-F: | CAGGTGGTGGATGATATTCTCGATG |
| *GGPPS*-011430-q-R: | ATTAGCCAAAGCAATCAACGGTG |
| *GGPPS*-029682-q-F: | CAGGTGGTGGATGATATTCTTGATG |
| *GGPPS*-029682-q-R: | GCAATCAAGGGAAGAGCCTTCTC |
| *GGPPS*-016696-q-F: | CAGGTGGTGGATGATATTTTGGAT |
| *GGPPS*-016696-q-R: | AATGGTACAATGGTGCAGCCC |
| *FPS*-009896-q-F: | AGTGCACTTGGTTGGTGCAT |
| *FPS*-009896-q-R: | ATTTACGGCAATCATACCAACC |
| *SS*-027479-q-F: | GCAGGACTTGTTGGATTAGGGT |
| *SS*-027479-q-R: | AACATGCGTGACTTTGGTATCTC |
| *DS*-000535-q-F: | CTAGCATAGCCCATGATGTTTGT |
| *DS*-000535-q-R: | TGAAGCAGTTCGTAAGGGTGTT |
| *CYP716A47*-011429-q-F: | AAGGTGGACGGGCTGAAAAA |
| *CYP716A47*-011429-q-R: | GGCGATTCCAATCCGTCTCT |
| *CYP716A53v2*-006374-q-F: | GGAGATGGACCTATGCCATTCAC |
| *CYP716A53v2*-006374-q-R: | TAGGAAGACCATTCTCCGGGAAG |
| *UGT74AE2*-011182-q-F: | TATCGTGGATGTATGGCGAATTG |
| *UGT74AE2*-011182-q-R: | GTACACAAGCTTTGAAAGGAACGC |
| *UGT74AE2*-024572-q-F: | GTTGTGGACGTATGGCAAACTG |
| *UGT74AE2*-024572-q-R: | CGTCGATATTCTTATCAGAGCTTCC |
| *UGT74AE2*-024577-q-F: | CGCTAAATATGTTGTGGACGTATGG |
| *UGT74AE2*-024577-q-R: | CGATATTCTTATCAGAGCTTCCACC |
| *UGT74AE2*-024582-q-F: | ATATGTTGTGGACGTGTGGCG |
| *UGT74AE2*-024582-q-R: | TATCAGAGCTTCCACCTTCACTCAT |
|  |  |
| *Promotor nested PCR amplification primers* |  |
| *proPnEXPA4*-F1 | TGAATAATCTCGCCGCAACGT |
| *proPnEXPA4*-F2 | GATCGAATTGAAGCTGCAAAAAGT |
| *proPnEXPA4*-F3 | GTGATAAGTTGTCCTCCTCCAATGG |
| *proPnEXPA4*-R | CACCAGATGGAGCAACATACCCA |
| *proPnGAP*-F1 | ACGTGGGTGTAGGAATCTGTTTG |
| *proPnGAP*-F2 | TACGGGGAAGAAGGTGTTATTAATG |
| *proPnGAP*-F3 | GTTTTTGGTGATTGAGACGCGT |
| *proPnGAP*-R | GAAAAAGTCGGGTGAAATCAAGAA |
| *proPnGGPPS*-F1 | GCCCAGCCTTAACATTGCCTAG |
| *proPnGGPPS*-F2 | GTGCCCAACTGCTTGATAAGTCG |
| *proPnGGPPS*-F3 | AGGCCGTGGCAAAGGATAAGAGA |
| *proPnGGPPS*-R | AGTTGAATTTACATGAATGGCCTGA |
| *proPnFPS-F1* | GCCTGTTTGTCAGATCTACTTCC |
| *proPnFPS-F2* | TATGGTTGCTTTCTATCGTTGAAGT |
| *proPnFPS-F3* | GGACGAAGATGGTGTAGTTGAAGAA |
| *proPnFPS-F4* | TCTCATAATTCATAAATGCTGCCAT |
| *proPnFPS-R* | TGCTGATTTTTGTTACCCGCTC |
| *proPnCYP716A47-F1* | TATCCCAACTAGTCTCTTTCCGCTGC |
| *proPnCYP716A47-F2* | TTCTATAACAACGCGGTTTTGAGT |
| *proPnCYP716A47-F3* | CATTGGGATGGAGAGTTGATGGT |
| *proPnCYP716A47-R* | TTAAAGTCGTCCGGTCCGGTC |
| *proPnCYP716A53v2-F1* | CACAGCCAAACCCAGTAGAGACGA |
| *proPnCYP716A53v2-F2* | AGGATGGCCCATAATTGGAGAAAC |
| *proPnCYP716A53v2-F3* | CAAGCTTGTTGTGTCCTGGTGGC |
| *proPnCYP716A53v2-R* | ATATCAGTCCTCTTCTCCCGCGA |
|  |  |
| *Bait construction primers* |  |
| *proGAP*-MBS-SacI-F | cGCCTAGTACTTTTCTTCATCCAAACTTCGGTCCTCCAACTGTGCATCCc |
| *proGAP*-MBS-XhoI-R | tcgagGGATGCACAGTTGGAGGACCGAAGTTTGGATGAAGAAAAGTACTAGGCgagct |
| *proGGPPS*-MBS-SacI-F | cTCTTTCTCCACATGCAAGTGTTTGTGACTTTGTGTTGAAGGGGGTATATAc |
| *proGGPPS*-MBS-XhoI-R | tcgagTATATACCCCCTTCAACACAAAGTCACAAACACTTGCATGTGGAGAAAGAgagct |
| *proGAP*-P1BS-SacI-F | cTGTTGAAGCTTATAAACCAAAATATTCAATAGAGTGATGAGAATc |
| *proGAP*-P1BS-XhoI-R | tcgagATTCTCATCACTCTATTGAATATTTTGGTTTATAAGCTTCAACAgagct |
| *proGGPPS*-P1BS-SacI-F | cCTACCGATTAGCTATTGTGCTTACATATGCATATAGCTCTCCGTAATTGc |
| *proGGPPS*-P1BS-XhoI-R | tcgagCAATTACGGAGAGCTATATGCATATGTAAGCACAATAGCTAATCGGTAGgagct |
| *proEXPA4*-P1BS-SacI-F | cGATTATACTTAAATTAATGAGAATATACATGATATCTTTGTTATTCGc |
| *proEXPA4*-P1BS-XhoI-R | tcgagCGAATAACAAAGATATCATGTATATTCTCATTAATTTAAGTATAATCgagct |
| *proCYP716A47*-P1BS-SacI-F | cAGGTACTAAGGAGGAGAGCAGATATTCTGTCCCTATTTTCTTTGc |
| *proCYP716A47*-P1BS-XhoI-R | tcgagCAAAGAAAATAGGGACAGAATATCTGCTCTCCTCCTTAGTACCTgagct |
| *proCYP716A53v2*-MBS-SacI-F | cAGGCAGCACTTTGAAGCCAAATGGTTTGTAATACTTCTCTCTc |
| *proCYP716A53v2*-MBS-XhoI-R | tcgagAGAGAGAAGTATTACAAACCATTTGGCTTCAAAGTGCTGCCTgagct |
| *proFPS*-MBS-SacI-F | cGCACATTCATATGTAAACAGTTGTTGGAGTGGTTAATATc |
| *proFPS*-MBS-XhoI-F | tcgagATATTAACCACTCCAACAACTGTTTACATATGAATGTGCgagct |
|  |  |
| *Prokaryotic expression primers* |  |
| *pMAL-PnPHL8-BamHI-F* | GAAGGATTTCAGAATTCGGATCCATGGCTCTACAGAATACACA |
| *pMAL-PnPHL8-BamHI-R* | CTGCAGGTCGACTCTAGAGGATCCTTATAGATGTTCATTGAATTG |
|  |  |
| *EMSA probes* |  |
| *pGAP-MBS-Bio-F* | GCCTAGTACTTTTCTTCATCCAAACTTCGGTCCTCCAACTGTGCATCC |
| *pGAP-MBS-Bio-R* | GGATGCACAGTTGGAGGACCGAAGTTTGGATGAAGAAAAGTACTAGGC |
| *pGAP-MBS-F* | GCCTAGTACTTTTCTTCATCCAAACTTCGGTCCTCCAACTGTGCATCC |
| *pGAP-MBS-R* | GGATGCACAGTTGGAGGACCGAAGTTTGGATGAAGAAAAGTACTAGGC |
| *pGAP-MBS-Mut-Bio-F* | GCCTAGTACTTTTCTTCATCCAAACTTCGGTCCTCAAAAAATGCATCC |
| *pGAP-MBS-Mut-Bio-R* | GGATGCATTTTTTGAGGACCGAAGTTTGGATGAAGAAAAGTACTAGGC |
| *pGGPPS-P1BS-Bio-F* | CTACCGATTAGCTATTGTGCTTACATATGCATATAGCTCTCCGTAATTG |
| *pGGPPS-P1BS-Bio-R* | CAATTACGGAGAGCTATATGCATATGTAAGCACAATAGCTAATCGGTAG |
| *pGGPPS-P1BS -F* | CTACCGATTAGCTATTGTGCTTACATATGCATATAGCTCTCCGTAATTG |
| *pGGPPS-P1BS -R* | CAATTACGGAGAGCTATATGCATATGTAAGCACAATAGCTAATCGGTAG |
| *pGGPPS-P1BS-Mut-Bio-F* | CTACCGATTAGCTATTGTGCTTAAAATTTTATATAGCTCTCCGTAATTG |
| *pGGPPS-P1BS-Mut-Bio-R* | CAATTACGGAGAGCTATATAAAATTTTAAGCACAATAGCTAATCGGTAG |
| *pCYP716A47-P1BS-Bio-F* | AGGTACTAAGGAGGAGAGCAGATATTCTGTCCCTATTTTCTTTG |
| *pCYP716A47-P1BS-Bio-R* | CAAAGAAAATAGGGACAGAATATCTGCTCTCCTCCTTAGTACCT |
| *pCYP716A47-P1BS -F* | AGGTACTAAGGAGGAGAGCAGATATTCTGTCCCTATTTTCTTTG |
| *pCYP716A47-P1BS -R* | CAAAGAAAATAGGGACAGAATATCTGCTCTCCTCCTTAGTACCT |
| *pCYP716A47-P1BS-Mut-Bio-F* | AGGTACTAAGGAGGAGAGCAAAATTTTTGTCCCTATTTTCTTTG |
| *pCYP716A47-P1BS-Mut-Bio-R* | CAAAGAAAATAGGGACAAAAATTTTGCTCTCCTCCTTAGTACCT |

**Supplementary Table 3. Retention times, precursor ions, product ions, declustering potentials and collision energies of 4 ginsenosides**

| Compound | Retention time (min) | Precursor ion (m/z) | Product ion (m/z) | Declustering potential (eV) | Collision energy (eV) |
| --- | --- | --- | --- | --- | --- |
| Ginsenoside Rg1 | 1.67 | 802.5 | 424.4 | 46.54 | 16.99 |
| Ginsenoside Re | 1.63 | 948.7 | 768.5 | 19.98 | 9.04 |
| Ginsenoside Rg1 | 2.05 | 1110.7 | 487.1 | 185.46 | 22.06 |
| Notoginsenoside R1 | 1.58 | 933.6 | 324.8 | 161.38 | 25.96 |

**Supplementary Table 4. Comparison of culture environments and techniques of *P. notoginseng* from different eras.**

|  | *Qing* dynasty | 1950s | Contemporary era(Medicine, 2019) |
| --- | --- | --- | --- |
| Climatic environment | “*Baise*…muggy and steamed from July to September”(Hua, 1967) | Annual average temperature, 20 ℃; annual precipitation, 1750-2000 mm(He and Deng, 1981) | annual average temperature, 15-17 ℃; annual precipitation, 900-1300 mm |
| Soil | Not mentioned | Loose and fertilized acid sandy loam, humus or improved clay (He and Deng, 1981) | Carbonate carbonite red soil, yellow argillaceous lateritic loam; loamy clay, pH5-6.5 |
| Illumination | “Grow in wooded valley without light”(Huang et al., 2007) | Light transmittance 40%-60%(Chen, 1958) | Light transmittance 8%-20% |
| Fertilization | Not mentioned | Phosphatic fertilizer combined with base fertilizer including animal manure, sod ash, oil bran, 1500 g-2000 g per Mu; topdressing 2-3 times, using plant ash 25-30 kg per Mu; dried human and animal excreta, 1000-1500 kg per Mu(Chen, 1958; He and Deng, 1981) | Farmyard manure as base fertilizer, 2500kg per Mu; topdressing 2 times, using farmyard manure 2500 kg per Mu, or compound fertilizer 15 kg per Mu, combined with potassium sulfate 10-15 kg per Mu. Nitrate nitrogen is forbidden to use |

**Supplementary References**

Chen, Z.Z. (1958). Review of *Panax notoginseng*. *Bulletin of Chinese Materia Medica* 4(7)**,** 224-230.

He, Z.X., and Deng, X.Q. (1981). Investigation on cultivation of *Panax notoginseng* in Guangxi and Yunnan (in Chinese). *Chin Tradit Herbal Drugs* 12(9)**,** 28-31.

Hua, B.S. (1967). *County annals of Baise.* Taibei: Chengwen Publishing House.

Huang, R.S., Yang, H.J., He, Z.J., Chen, C.J., Li, Z.W., and Zhang, C.L. (2007). Textual research on the origin in areas of *Panax notoginseng*. *Lishizhen Medicine and Materia Medica Research* 18(7)**,** 1610-1611.

Medicine, C.A.o.C. (2019). "Daodi herbs (in Chinese)", in: *Part 118: Sanqi.* (Beijing: China Standard Press).

Xu, N. (2016). *Research of the factors influencing commodity quality of Notoginseng Radix et Rhizoma.* Kunming University of Science and Technology.
